# Genetics of chilling response at early growth stage in rice: a recessive gene for tolerance and importance of acclimation

**DOI:** 10.1093/aobpla/plad075

**Published:** 2023-11-08

**Authors:** Akhil Ranjan Baruah, Hiroaki Bannai, Yan Meija, Ayumi Kimura, Haruka Ueno, Yohei Koide, Yuji Kishima, Jiwan Palta, Jun Kasuga, Masayuki P Yamamoto, Kazumitsu Onishi

**Affiliations:** Department of Agricultural Biotechnology, Assam Agricultural University, Jorhat-13, Assam, India; Research Faculty of Agriculture, Hokkaido University, Sapporo, Hokkaido 060-8589, Japan; Department of Agro-Environmental Science, Obihiro University of Agriculture and Veterinary Medicine, Obihiro, Hokkaido 080-8555, Japan; Department of Agro-Environmental Science, Obihiro University of Agriculture and Veterinary Medicine, Obihiro, Hokkaido 080-8555, Japan; Department of Agro-Environmental Science, Obihiro University of Agriculture and Veterinary Medicine, Obihiro, Hokkaido 080-8555, Japan; Department of Agro-Environmental Science, Obihiro University of Agriculture and Veterinary Medicine, Obihiro, Hokkaido 080-8555, Japan; Research Faculty of Agriculture, Hokkaido University, Sapporo, Hokkaido 060-8589, Japan; Research Faculty of Agriculture, Hokkaido University, Sapporo, Hokkaido 060-8589, Japan; Department of Horticulture, University of Wisconsin-Madison, 490 Moore Hall, 1575 Linden Drive, Madison, WI 53706, USA; Department of Agro-Environmental Science, Obihiro University of Agriculture and Veterinary Medicine, Obihiro, Hokkaido 080-8555, Japan; Faculty of Science, Academic Assembly, University of Toyama, 3190 Gofuku, Toyama 930-8555, Japan; Department of Agro-Environmental Science, Obihiro University of Agriculture and Veterinary Medicine, Obihiro, Hokkaido 080-8555, Japan

**Keywords:** Acclimation, breeding, chilling tolerance, NLR gene, rice

## Abstract

Low-temperature adaptation in rice is mediated by the ability of a genotype to tolerate chilling temperatures. A genetic locus on chromosome 11 was analysed for chilling tolerance at the plumule stage in rice. The tolerant allele of A58, a *japonica* landrace in Japan, was inherited as a recessive gene (*ctp-1*^A58^), whereas the susceptible alleles from wild rice (*Ctp-1*^W107^) and modern variety (*Ctp-1*^HY^) were the dominant genes. Another recessive tolerant allele (*ctp-1*^Silewah^) was found in a *tropical japonica* variety (Silewah). Fine-mapping revealed that a candidate gene for the *ctp-1* locus encoded a protein similar to the nucleotide-binding domain and leucine-rich repeat (NLR) protein, in which frameshift mutation by a 73 bp-deletion might confer chilling tolerance in *ctp-1*^A58^. Analysis of near-isogenic lines demonstrated that *ctp-1*^A58^ imparted tolerance effects only at severe chilling temperatures of 0.5 °C and 2 °C, both at plumule and seedling stages. Chilling acclimation treatments at a wide range of temperatures (8 °C–16 °C) for 72 h concealed the susceptible phenotype of *Ctp-1*^W107^ and *Ctp-1*^HY^. Furthermore, short-term acclimation treatment of 12 h at 8 °C was enough to be fully acclimated. These results suggest that the NLR gene induces a susceptible response upon exposure to severe chilling stress, however, another interacting gene(s) for acclimation response could suppress the maladaptive phenotype caused by the *Ctp-1* allele. This study provides new insights for the adaptation and breeding of rice in a low-temperature environment.

## Teaser

This paper showed that a recessive gene (ctp-1) conferred tolerance to severe chilling stress and its candidate gene was nucleotide-binding domain and leucine-rich repeat (NLR) gene, known as disease resistance gene. The role of acclimation response, which enhances chilling tolerance by pre-exposure to mild stress temperature before severe stress, has been mostly ignored in rice breeding; however, we demonstrated that gene(s) other than the intrinsic tolerance gene (the ctp-1 locus) could contribute to improvement of chilling tolerance.

## Introduction

Tolerance to low temperatures is a critical determinant that allows plant species to adapt to diverse environmental cues (Levitt 1980). Based on the plant’s response, low-temperature tolerance is classified into two categories: chilling (above 0 °C) and freezing (below 0 °C). Our understanding of the genetic and molecular mechanisms underlying low-temperature tolerance has been supplemented by intensive research on freezing tolerance in the model plant, Arabidopsis (*Arabidopsis thaliana*) (Ding *et al.* 2019). Tolerance to chilling temperature is also equally important in plants because various degrees of chilling temperatures during the spring to summer seasons often affect germination, vegetative growth and reproductive success (Levitt 1980; Graham and Patterson 1982). Chilling tolerance mediates diverse physiological and developmental processes and metabolic adjustments, which are affected by the degree of stress (temperature and duration), organ type, developmental stage and other environmental conditions, such as humidity and light (Levitt 1980; Hussain *et al.* 2018).Our knowledge on genetic and physiological mechanisms of the response to chilling temperature remains elusive for most plant species, including rice (Guo *et al.* 2018; Ding *et al.* 2019).

Asian rice (*Oryza sativa*) is an important cereal crop cultivated under a wide range of environmental conditions, from tropical to subarctic zones. Although its origin is debatable, cultivated rice has diverged into subspecies through repeated hybridization and distribution across Asia (Huang *et al.* 2012; Wang *et al.* 2018). Although rice is a chilling-sensitive plant, a wide range of genetic variations exist for chilling tolerance among its germplasms (Li *et al.* 2022). Chilling stress induces diverse responses (or damage) in rice plants during all growth stages, from germination to maturity, such as delayed germination and growth, chlorosis (yellowing) and necrosis (browning) of leaves, withering of seedlings and spikelet sterility, which ultimately lead to a decline in yield (Li *et al.* 2022).

Over the last two decades, the genetic basis of natural variation in chilling tolerance in the rice germplasm has been investigated. These studies have revealed the genetic architecture of numerous quantitative trait loci (QTLs) for traits throughout the rice genome (Andaya and Mackill 2003; Zhi-Hong *et al.* 2005; Baruah *et al.* 2009; Koseki *et al.* 2010; Suh *et al.* 2011; Wang *et al.* 2011; Yang *et al.* 2013; Mao *et al.* 2015; Xiao *et al.* 2015; Jiang *et al.* 2017; Najeeb *et al.* 2020). Our understanding of the mechanisms and evolution of chilling tolerance in rice has been enhanced by recent molecular characterization of various chilling tolerant genes (Liu *et al.* 2013, 2018; Ma *et al.* 2015; Zhang *et al.* 2017; Zhao *et al.* 2017; Xiao *et al.* 2018; Mao *et al.* 2019; Zheng *et al.* 2022; Li *et al.* 2023); however, the tolerance to chilling temperature usually shows complex inheritance, for which effective genes or QTLs differ depending on genetic background, developmental stages and organs, chilling temperature and duration of stress exposure (Guo *et al.* 2018; Li *et al.* 2022). Therefore, further efforts to identify and characterize chilling tolerance genes are required to breed new varieties with higher adaptability suitable for cold climatic conditions.

Cold-tolerant species such as wheat, barley and Arabidopsis are able to acclimate to sub-optimal temperatures that induce freezing tolerance response in these plants (Thomashow 1999; Ding *et al.* 2019). The acclimation response involves drastic physiological changes that are crucial for the survival of plant species grown in high latitudinal regions during the winter season (Thomashow 1999; Ding *et al.* 2019). However, the importance of acclimation ability for chilling-sensitive plants, including rice, has not yet been critically analysed. In rice, most studies involving the evaluation of genetic variation and genetic control for chilling tolerance have focused on understanding the degree of intrinsic tolerance without acclimation treatment. Information on chilling acclimation ability and its role in improving chilling tolerance in rice is limited (Baruah *et al.* 2011; Mao and Chen 2012; Xie *et al.* 2012; Cen *et al.* 2018). Examination of the involvement of major QTLs detected under controlled stress conditions in acclimation response is critical.

Previously, *Oryza sativa* ssp. *japonica* strains were reported to show higher chilling tolerance than *O. sativa* ssp. *indica* and wild rice (*O. rufipogon*) strains, and three QTLs were detected for chilling tolerance at the plumule stage using recombinant inbred lines developed from a cross between A58, a chilling-tolerant *japonica* landrace, and W107, an annual wild rice strain (Baruah *et al.* 2009). One of the three QTLs, *qCTP11* (*quantitative trait locus for chilling tolerance at the plumule stage-11*), was mapped on the long arm of chromosome 11 (Baruah *et al.* 2009). Furthermore, chilling tolerance was found to be significantly enhanced by acclimation, and genetic variations in acclimation ability reportedly exist in Asian cultivated rice and its wild progenitors (Baruah *et al.* 2011). The objective of the present study was to further explore the genetic control of chilling tolerance and chilling acclimation ability. Our results showed that chilling tolerance is controlled by a single recessive gene (*ctp-1*) at *qCTP11*. Fine-mapping of the *ctp-1* locus suggested that a loss-of-function mutation in the gene encoding a protein similar to the nucleotide-binding domain and leucine-rich repeat (NLR) protein was responsible for chilling tolerance. Furthermore, our results clearly showed that other gene(s) than the *ctp-1* locus could be responsible for acclimation ability, implying a caution for breeding intrinsic chilling tolerance without acclimation treatment.

## Materials and Methods

### Plant materials

Two backcross populations or near isogenic lines (NILs) for *qCTP11* were developed in two different ssp. *japonica* (A58 and ‘Hoshinoyume’) background by backcrossing. A58 is a landrace and ‘Hoshinoyume’ (HY) is an elite variety. Both are derived from Hokkaido Island, Japan (41 °N–45 °N), which is one of the northernmost rice-cultivating regions of the world. To develop NIL, the susceptible allele of W107 (an *O. rufipogon* strain from India) was introduced into A58 (cold-tolerant strain) using marker-assisted selection (MAS). Previously, *qCTP11* was mapped in the BC_4_ generation (Baruah *et al.* 2009). Three more rounds of backcrossing were performed to develop NILs in the A58 background. HY exhibited a chilling-susceptible phenotype, as shown in the ‘Results’ section. The A58 allele was introgressed into HY by backcrossing and was confirmed using MAS.

To evaluate the genetic variations in chilling tolerance in Hokkaido Island, 11 varieties or breeding lines were used. The plant materials used in this study were provided by the Plant Breeding Laboratory of Hokkaido University, Japan, and Hokkaido Kamikawa Agricultural Experimental Station in Pippu, Hokkaido, Japan.

### Chilling tolerance assays and acclimation treatment

**Figure 1.**
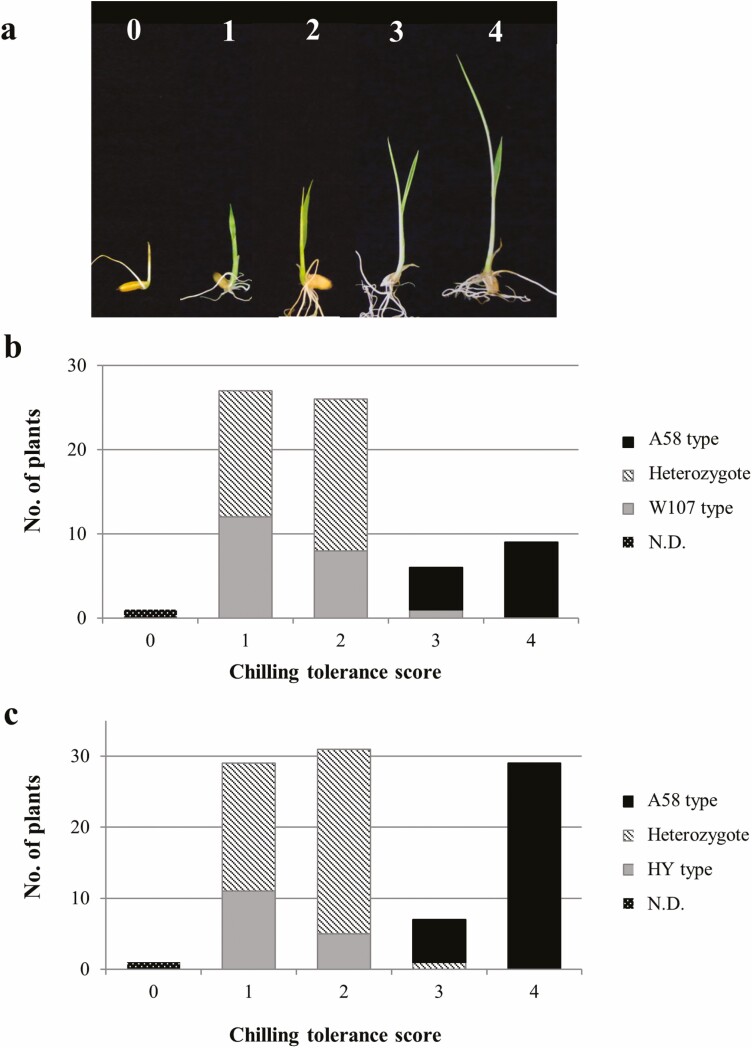
Segregation of chilling tolerance in two backcross populations for *qCTP11.* (A) Chilling tolerance score for evaluation of the degree of tolerance. A score of ‘0’ indicated that the plants were dead, and plumules turned brown immediately after the end of chilling treatment. A score of ‘4’ indicated that plants continued to grow rapidly with normal leaf colour (Baruah *et al.* 2009). (B) A58 background BC_6_F_2_, (C) HY background BC_2_F_2_. A58, HY and W107-type indicate the homozygotes of A58, HY and W107 derived allele, respectively, for a flanking marker (P054 for A58 background BC_6_F_2_, RM1219 for HY background BC_2_F_2_). N.D. indicates not determined.

Chilling tolerance at the plumule stage was evaluated using two methods (petri dish and small cup methods). The petri dish method was as described by Baruah *et al.* (2009). In this method, seeds were germinated in petri dishes at 30 °C to obtain plumules. Ten or more seeds with plumule length of 1.0–1.5 cm were selected and exposed to chilling stress (0.5 °C) for 2 d in the dark in an incubator (MIR-254, Panasonic Healthcare Co., Ltd., Gunma, Japan). They were then transferred to a growth chamber (BIOTRON LPH200, Nippon Medical & Chemical Instruments Co. Ltd., Osaka, Japan) set at 26 °C with 300 μmol·m^−2^·s^−1^ light intensity and a 14 h light/10 h dark cycle. Lids of the petri dishes were removed and arranged in plastic boxes, which were sealed with transparent plastic wraps to prevent desiccation. The chilling tolerance among the lines was determined after 6–8 d of chilling treatment, based on the degree of cold-induced injuries in the plumules, using scores of 0 (susceptible) to 4 (tolerant) (Fig. 1A). A score of ‘0’ indicated that the plants were dead, and plumules turned brown immediately after the end of chilling treatment, whereas a score of ‘4’ indicated that plants continued to grow rapidly with normal leaf colour (Baruah *et al.*2009).

The small cup method was used to assess the continuous differences in degree of tolerance. Seeds of each strain were germinated on a filter paper in a petri dish at 30 °C for 1–2 d. Eight germinated seeds were planted in a small plastic cup (6.5 cm in diameter, 3.5 cm in height) filled with 30 mL soil (15 mL culture soil mixed with 15 mL vermiculite). The small cup with lid was kept at 30 °C for one day in the incubator until plumules grew approximately 1 cm above the soil surface. Then, small cup with lid was exposed to chilling stress in dark in the incubator (0.5 °C, 2 °C, 4 °C, 6 °C or 8 °C for 2 d). After the chilling treatment, small cup without lid was kept in the growth chamber for 10 d, set at 25 °C with 300 μmol·m^−2^·s^−1^ light intensity and a 14 h light/10 h dark cycle. As a control, a small cup for each strain was kept in the same growth chamber without chilling treatment for 10 d. Plant height (length from the soil surface to the leaf tip) was measured, and the percentage of plant height (chilling treatment/control × 100) was used to measure the degree of chilling tolerance. About 24 plumules (3 cups) were evaluated for each treatment of each strain.

To evaluate chilling tolerance at the seedling stage, eight seedlings were grown in a small cup without lid kept in a growth chamber set at 25 °C with 300 μmol·m^−2^·s^−1^ light intensity and 14 h light/10 h dark cycle for 2 weeks. Then, chilling stress (0.5 °C, 2 °C, 4 °C, 6 °C or 8 °C for 2 d) was exposed under dark conditions in the incubator. After the chilling treatment, the small cup was kept in the growth chamber (25 °C, 14 h day-length) for 2 weeks. As a control, small cup was kept in the same growth chamber without chilling treatment for 2 weeks. The length from the soil surface to the newly emerged leaf tip after the chilling treatment was measured as the plant height. The percentage of plant height (chilling treatment/control × 100) was used to measure the degree of tolerance. About 24 seedlings (three cups) were evaluated for each treatment of each strain.

The effect of acclimation was examined at the plumule stage using the small cup method. Before chilling treatment, plumules were subjected to acclimation treatment at various temperatures (6 °C, 8 °C, 10 °C¸12 °C, 14 °C and 16 °C) for 72 h and different durations (12, 24, 48 and 72 h) at 8 °C in small cups with lids under dark in the incubator. Then, small cup with lid was exposed to chilling stress in the dark in the incubator (0.5 °C for 2 d). After the chilling treatment, the small cup was kept (25 °C, 14 h day-length) for 2 weeks. As a control, small cup was kept in the same growth chamber for 2 weeks without chilling and acclimation treatments. The degree of chilling tolerance (percentage of plant height) of acclimated plants was evaluated using the methods, as described above.

### Assessing mode of inheritance for *qCTP11*

To understand the mode of inheritance of *qCTP11*, chilling tolerance was examined in BC_6_F_2_ and BC_2_F_2_ populations from A58 and HY backgrounds, respectively. The F_2_ population derived from Joukei-04501 (J501) × HY was used to investigate the segregation of the chill-tolerant gene of J501. Chilling tolerance was examined using the petri dish method as described above. After evaluation of the phenotype, the plumule and root of each plant were sampled for DNA extraction. The association between the chilling tolerance score and genotype, as revealed by molecular markers tightly linked to *qCTP11*, was assessed for each population. Fifty-nine to 98 plants were evaluated in each population.

### Fine mapping of *qCTP11* and sequencing of candidate region

Recombinant plants were selected from 5092 segregating populations (BC_4_F_2_ or later generations). First, the recombinant plants in the QTL region (between markers J03 and F10) were selected from the segregating population, and homozygotes for recombined chromosomal segments were selected in progenies. After self-pollination of homozygous lines, chilling tolerance was evaluated using the Petri dish method, as described above.

Genomic DNA was isolated from plumules or seedlings according to the method of Monna *et al.* (2002) with slight modifications. For fine mapping, simple sequence repeat or sequence-tagged site markers reported by Baruah *et al.* (2009) were used [Supporting Information—Table S1]. For sequencing, genomic DNA was isolated from plumules or seedlings using DNeasy Plant Kits (QIAGEN, Germantown, MD, USA) according to the manufacturer’s protocol. Two sequence-based SNP markers (SNPa and SNPb) were also used to aid in fine mapping [Supporting Information—Table S2]. To obtain specific PCR products for the candidate regions, a nested PCR method was used, as summarized in Supporting Information—Fig. S2 and Supporting Information—Table S2. As described in ‘Results’ section, the candidate region was duplicated in ‘Nipponbare’ reference genome sequence. Therefore, region-specific primers (2011Long-L1 and NcpsE-R) were used for the first PCR and the PCR products of the second PCR were used for sequencing. The PCR products were purified on a Labo Pass™ Gel using a DNA Purification Reagent Kit (Hokkaido System Science Ltd., Sapporo, Japan) and sequenced by the chain termination method using a 3730 XL DNA Analyzer (Applied Biosystems, Foster, CA, USA).

## Results

### Recessive inheritance of chilling tolerance

To determine whether the tolerant allele at *qCTP11* was dominant or recessive, backcross populations from two different genetic backgrounds (A58 and HY) were analysed. The segregation pattern of susceptible (score 0 to 2) and resistant (score 3 and 4) F_2_ plants was fitted to the 3:1 ratio (*χ*^2^ = 0.39, *P* = 0.53) in the A58 background BC_6_F_2_ population (Fig. 1B), but the segregation pattern significantly deviated from the 3:1 ratio (*χ*^2^ = 7.20, *P* < 0.01) in the HY background BC_2_F_2_ population (Fig. 1C). Analyses of the molecular markers tightly linked to *qCTP11* (P054 for A58 background BC_6_F_2_, RM1219 for HY background BC_2_F_2_) showed that plumules with scores of 3 and 4 had A58-derived alleles in homozygous conditions, except for one plant in each population. In contrast, plumules with scores of 1 and 2 were homozygous for the W107 or HY allele and heterozygous. These results showed that the tolerant allele at *qCTP11* behaved as a single recessive gene in two genetic backgrounds. Therefore, the tolerant allele from A58 was designated as *ctp-1*^A58^ and the susceptible alleles from W107 and HY were denoted as *Ctp-1*^W107^ and *Ctp-1*^HY^, respectively.

### Genetic variation in chilling tolerance in varieties grown in the northernmost region for rice cultivation

**Figure 2.**
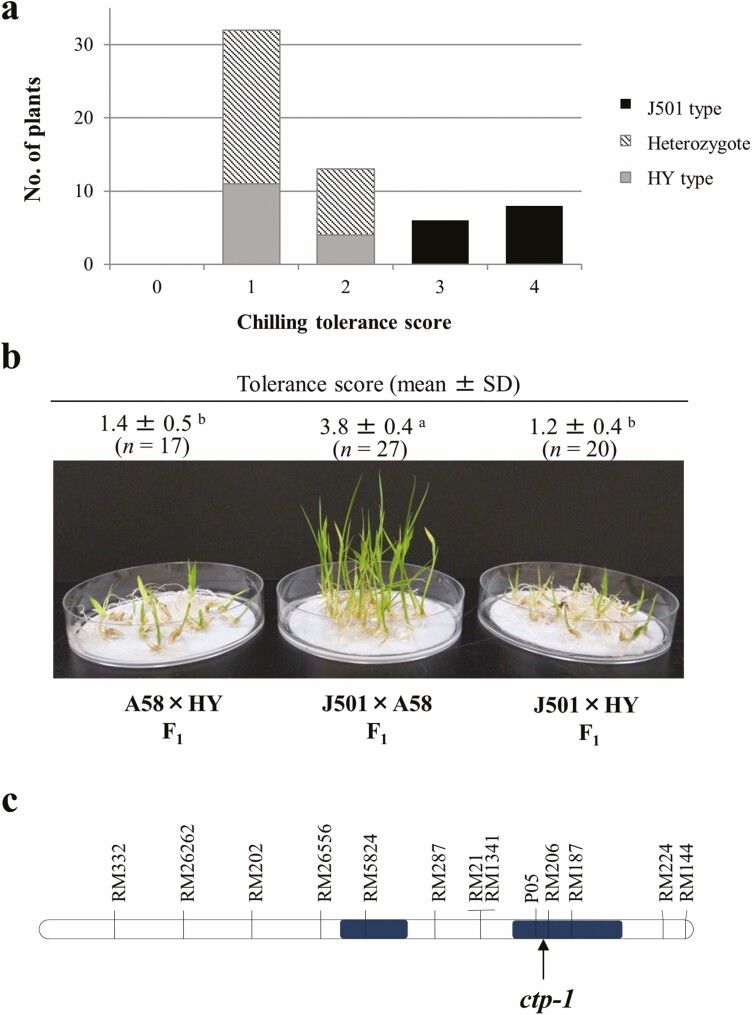
Analysis for chilling tolerance gene of J501. (A) Segregation of chilling tolerance in F_2_ plants from J501 × HY. J501 and HY-type indicate the homozygote of J501 and HY-derived allele, respectively, for a flanking marker (C189). (B) Allelism test for chilling tolerance alleles at the *ctp-1* locus between A58 and J501. Different letters indicate the significant difference between crosses (Tukey–Kramer test, *P* < 0.05). (C) Genomic regions of chromosomal segments of ‘Silewah’ introduced into J501 (Filled boxes).

The genetic variation in chilling tolerance at the plumule stage in Hokkaido varieties and breeding lines was evaluated. Among the 11 strains examined, only one breeding line, Joukei-04501 (J501), which was reported to possess high chilling tolerance at the booting stage, also exhibited tolerance comparable to that of A58, and these two strains had significantly higher chilling tolerance than the other strains (Supporting Information—Fig. S1). To determine whether the *ctp-1* locus was also associated with chilling tolerance of J501, F_2_ plants from the cross between J501 and HY were evaluated using molecular marker (C189) tightly linked to the *ctp-1* locus (Fig. 2A). The segregation pattern of susceptible (score 0 to 2) and resistant (score 3 and 4) F_2_ plants revealed a 3:1 ratio (*χ*^*2*^ = 0.051, *P* = 0.82), that is, plumules with scores 3 and 4 had a homozygous J501 allele and those with scores 1 and 2 were either homozygous for the HY allele or heterozygous. This result indicated that the recessive gene tightly linked to the marker C189 conferred chilling tolerance of J501.

Recessive inheritance of chilling tolerance permits the performance of an allelism test between *ctp-1*^A58^ and the tolerance gene of J501. *F*_1_ plants from the cross between A58 and J501 showed a tolerant phenotype, whereas *F*_1_ plants from both A58 × HY and J501 × HY showed a susceptible phenotype (Fig. 2B). This result indicated that the tolerant gene of J501was allelic to *ctp-1*^A58^. The breeding line, J501 was developed from the cross between Hokkaido varieties and a tropical *japonica* variety, ‘Silewah’ that is known to have high chilling tolerance at the booting stage (Satake and Toriyama 1979; Ishiguro *et al.* 2014). J501 was reported to carry chromosomal segments introgressed in the long arm of chromosome 11 from ‘Silewah’ (Mori *et al.* 2011). Analysis using molecular markers linked to the *ctp-1* locus revealed that J501 should have an allele at the *ctp-1* locus (*ctp-1*^Silewah^) derived from ‘Silewah’ (Fig. 2C).

### Fine mapping of *ctp-1* and elucidation of candidate gene

**Figure 3.**
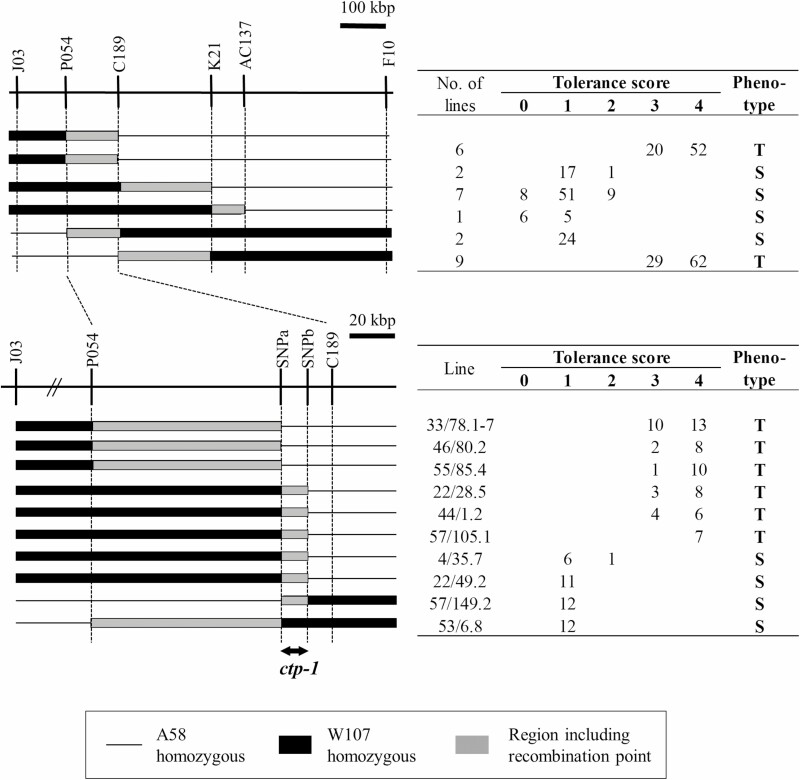
Fine-mapping of *ctp-1*. ‘T’ and ‘S’ indicate tolerant and susceptible phenotypes, respectively. Graphical genotypes were shown on a physical map based on the reference genome sequence of cv. ‘Nipponbare’.

Fine mapping was conducted in 5092 backcrossed segregating populations (BC_4_F_2_ or later generations). Previously, the candidate region of the *ctp-1* locus was delimited to 778 kbp, spanning F10 and J03 (Baruah *et al.* 2009). Recombinant selection between markers J03 and F10 and analysis using additional markers allowed delimiting the candidate region of *ctp-1* to approximately 12 kbp between SNPa and SNPb, where six recombinants were identified (Fig. 3).

**Figure 4.**
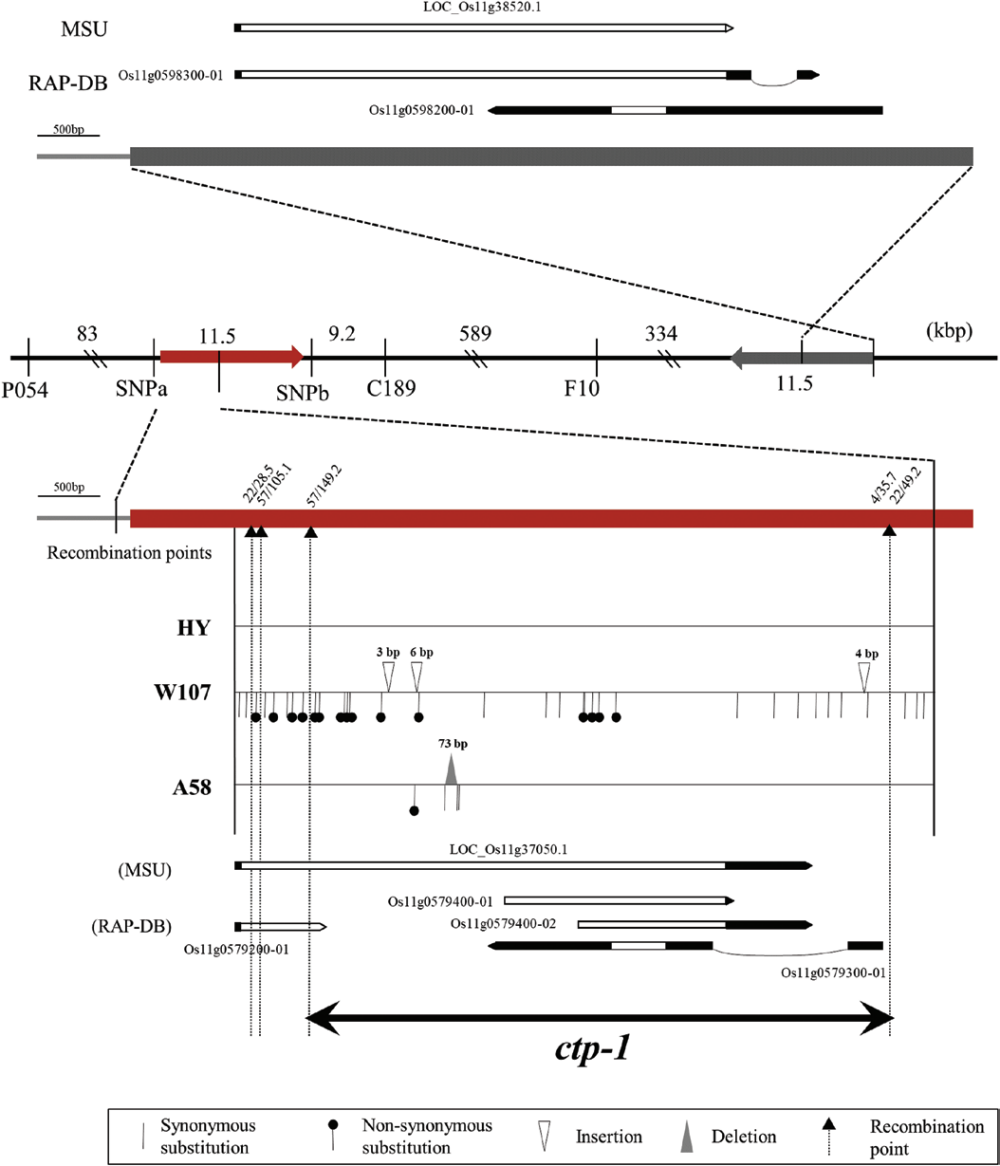
Candidate region of the *ctp-1* locus. Physical map is based on the reference genome sequence of cv. ‘Nipponbare’. Arrows on the physical map indicate the duplicated regions of approximately 11.5 kbp. Recombination points of five recombinants are indicated on the physical map. The predicted genes were as in the MSU Rice Genome Annotation Project (http://rice.uga.edu/) and Rice Annotation Project (RAP)-DB database (https://rapdb.dna.affrc.go.jp/). The sequences of A58, W107 (A58*Ctp-1*^W107^) and HY are compared with the reference genome sequence of cv. ‘Nipponbare’.

A survey of the reference rice genome sequence of cv. ‘Nipponbare’ revealed that the 11.5 kbp chromosomal segments, which duplicated at about 950 kbp downstream of chromosome 11, was included in the candidate region (Fig. 4). Sequence analysis of recombinant plants and parental strains (A58 and A58*Ctp-1*^W107^) revealed that recombination occurred in the 5.5 kbp region of the 11.5 kbp chromosomal segments. The results indicated that the *ctp-1* locus was delimited into 5044 bp region on the reference rice genome sequence (Fig. 4), which included one candidate gene (LOC_Os11g37050.1) in the MSU Rice Genome Annotation Project (http://rice.uga.edu/) database and four candidate genes (Os11g0579200-01, Os11g0579400-01, Os11g0579400-02, and Os11g0579300-01) in The Rice Annotation Project database (RAP-DB; https://rapdb.dna.affrc.go.jp). By comparing with reference genome sequence of ‘Nipponbare’, 73 bp deletions and three SNPs were detected in *ctp-1*^A58^, whereas 33 SNPs and three insertions were observed in *Ctp-1*^W107^ (Fig. 4). *Ctp-1*^HY^ had no mutation as compared to ‘Nipponbare’ reference genome sequence. Therefore, only 73 bp deletions and four SNPs in *ctp-1*^A58^ clearly distinguished the tolerant allele from the susceptible alleles (*Ctp-1*^W107^ and *Ctp-1*^HY^). Among the five annotated genes in the candidate region, three genes should have partial ORFs of LOC_Os11g37050.1 because Os11g0579200-01 has no stop codon and Os11g0579400-01 and Os11g0579400-02 have no start codon. The 73 bp deletion and three SNPs in *ctp-1*^A58^ were located in the ORF of LOC_Os11g37050.1, and downstream of Os11g0579300-01 (Fig. 4). The 73 bp deletion could be a loss-of-function variant due to a frameshift in LOC_Os11g37050.1 [Supporting Information—Fig. S3 and Supporting Information—Fig. S4A]. Although many synonymous and non-synonymous substitutions, as well as 3 and 6 bp indels, were observed between *Ctp-1*^W107^ and *Ctp-1*^HY^, proteins of 1420 and 1423 amino acids could be translated in *Ctp-1*^W107^ and *Ctp-1*^HY^, respectively [Supporting Information—Fig. S4B]. Considering that *ctp-1*^A58^ was a recessive allele and *Ctp-1*^W107^ and *Ctp-1*^HY^ were dominant alleles, LOC_Os11g37050.1 concluded to be the most likely candidate gene for the *ctp-1* locus. According to the annotation database, LOC_Os11g37050.1 encodes a protein similar to the nucleotide-binding domain and leucine-rich repeat (NLR) protein.

### The effects of *ctp-1* on the chilling tolerance at various temperature stress

**Figure 5.**
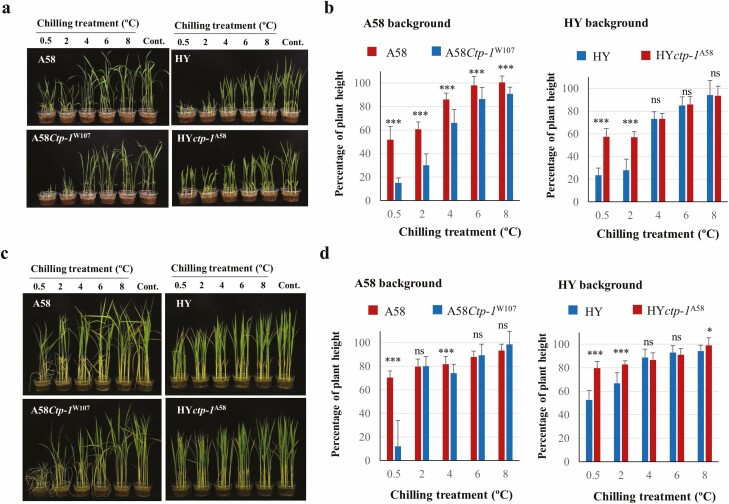
Effect of the *ctp-1* locus under different chilling temperature conditions at the plumule (A and B) and seedling stage (C and D) in two genetic backgrounds. Plant height 10 d after chilling treatment at plumule stage (A) and 2 weeks after chilling treatment at seedling stage (C). The degree of chilling tolerance at plumule (B) and seedling (D) stages was evaluated as the percentage of plant height, as described in ‘Materials and Methods’ section. ***, ** and * indicate significant differences between the NIL and its recurrent parent at the 0.1 %, 1 % and 5 % levels, respectively. ns indicates non-significant.

The effects of the *ctp-1* locus under various chilling temperature regimes at the plumule stages were evaluated using an NIL for *Ctp-1*^W107^ in the A58 background (A58*Ctp-1*^W107^) and an NIL for *ctp-1*^A58^ in the HY background (HY*ctp-1*^A58^) (Fig. 5A and B). With the decrease in chilling temperature, the plant height gradually decreased by 10 d after chilling treatment for all lines (Fig. 5A). At temperatures 4 °C and above, the differences in the degree of chilling tolerance (percentage of plant height) between genotypes carrying *ctp-1*^A58^and *Ctp-1*^W107^ or *Ctp-1*^HY^ were not significant or relatively small. At 2 °C and 0.5 °C, genotypes carrying *Ctp-1*^W107^ and *Ctp-1*^HY^ revealed chilling susceptible phenotypes showing a severe reduction in plant height, indicating that allelic differences in genetic effects appeared only at severe chilling stress lower than 2 °C.

To examine whether *ctp-1*^A58^ confers chilling tolerance at the seedling stage, 2-week-old seedlings were exposed to chilling stress (Fig. 5C and D). The plant height and the degree of chilling tolerance (percentage of plant height) showed a similar tendency 2 weeks after chilling treatment as shown in plumule stage, whereas chilling susceptible phenotype was shown only at 0.5 °C in A58*Ctp-1*^W107^. These results indicated that the *ctp-1* locus was involved in chilling response at both plumule and seedling stages. J501 showed a similar level of tolerance at plumule and seedling stages as that of A58 (Supporting Information—Fig. S5).

### Susceptible phenotype of *Ctp-1* is concealed by acclimation

**Figure 6.**
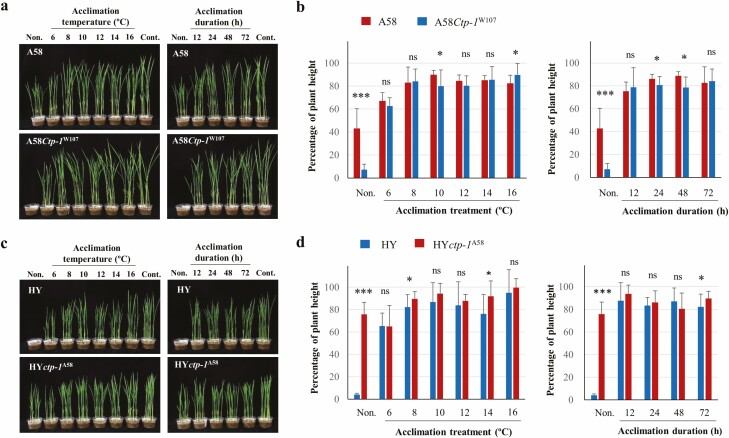
Effect of acclimation temperature and duration on NILs of the *ctp-1* locus under A58 (A and B) and HY (C and D) backgrounds. Before chilling treatment, plumules were subjected to acclimation treatment at various temperatures (6 °C, 8 °C, 10 °C¸12 °C, 14 °C and 16 °C) for 72 h and different durations (12, 24, 48 and 72 h) at 8 °C. Two weeks after chilling treatment (0.5 °C for 2 d), the degree of chilling tolerance (the percentage of plant height) of acclimated plants was evaluated, as described in ‘Materials and Methods’ section. *** and * indicate significant differences between the NIL and its recurrent parent at the 0.1 % and 5 % levels, respectively. ns indicates non-significant.

Previously, acclimation treatment (8 °C for 72 h) was reported to significantly increase chilling tolerance in rice (Baruah *et al.* 2011). Although A58 increased chilling tolerance by acclimation treatment, W107 (with *Ctp-1*^W107^allele), showed no or lower acclimation ability (Baruah *et al.* 2011). To examine whether susceptible allele reduce both intrinsic chilling tolerance and acclimation ability, the effects of acclimation under different acclimation temperature regimes and treatment durations were examined in NILs with two genetic backgrounds. Acclimation temperatures varied from 6 °C to 16 °C when duration was kept constant at 72 h, or duration of acclimation treatment varied from 12 h to 72 h when temperature was kept constant at 8 °C (Fig. 6). Although A58*Ctp-1*^W107^and HY revealed chilling susceptible phenotypes under chilling stress (0.5 °C, 2 d) without acclimation, acclimation treatments significantly increased the degree of chilling tolerance (percentage of plant height). Significant differences in degree of tolerance between A58 and A58*Ctp-1*^W107^, HY and HY*ctp-1*^A58^ were not observed across all acclimation treatments (*P* = 0.01), except for few (10 °C and 16 °C in A58/A58*Ctp-1*^W107^ and 8 °C and 14 °C in HY/HY*ctp-1*^A58^ at *P* = 0.05). These results indicate that susceptible phenotype of *Ctp-1* was concealed by acclimation and the *ctp-1* locus was not responsible for the acclimation response. Regarding the effects of acclimation temperature on chilling tolerance, the degree of chilling tolerance (percentage of plant height) was slightly lower at 6 °C than at any other temperature, whereas no large difference was observed in both NILs, HY and A58, at ≥8 °C. The duration of acclimation treatments from 12 h to 72 h detected a similar degree of chilling tolerance, indicating that 12 h at 8 °C was enough to be fully acclimated at plumule stage.

## Discussion

### Genetic system for chilling tolerance by a single recessive gene *ctp-1*

The genetic architecture of the response to low temperature is complex, involving multiple genes with diverse effects depending on environmental factors and developmental stages (Guo *et al.* 2018; Ding *et al.* 2019). Regarding chilling tolerance at the early growth stages in rice, previous QTL analysis and genome-wide association studies detected a number of QTLs with major and minor effects throughout rice genome (Andaya and Mackill 2003; Zhi-Hong *et al.* 2005; Baruah *et al.* 2009; Koseki *et al.* 2010; Suh *et al.* 2011; Wang *et al.* 2011; Yang *et al.* 2013; Mao *et al.* 2015; Xiao *et al.* 2015; Jiang *et al.* 2017; Najeeb *et al.* 2020). Present results demonstrate that *ctp-1* has contributed to chilling tolerance under severe chilling stress (≤2 °C) at the plumule and seedling stages, whereas most of the reported genes or QTLs in previous studies were evaluated under various chilling temperatures between 4 °C and 12 °C. This implies that different physiological and molecular mechanisms might be involved depending on the temperature profiles. Other environmental and developmental factors, such as duration of stress, stages or organs exposed to stress, humidity, light conditions and their interactions, should also affect the expression of genetic factors for chilling response (Levitt 1980; Hussain *et al.* 2018).

For the *ctp-1* locus, two tolerant alleles (A58 and Silewah) and two susceptible alleles (W107 and HY) were identified. Fine mapping revealed that the candidate gene of the *ctp-1* locus is LOC_Os11g37050.1, and detected a number of polymorphic sites, including amino acid changes between susceptible alleles (HY and W107); however, only the frameshift mutation by the 73 bp deletion clearly differed between the tolerant (recessive) allele of A58 and the susceptible (dominant) alleles of W107 and HY. For the Silewah allele (*ctp-1*^Silewah^), PCR amplification of the candidate region failed despite several trials. The available public sequence database of the rice diversity panel (Tanaka *et al.* 2020, https://ricegenome-corecollection.dna.affrc.go.jp/) showed no or low depth of next-generation sequencing data in the region including LOC_Os11g37050.1 for most strains of the *indica*, *aus* and *tropical japonica*. Therefore, structural variations might have occurred in the target region during the evolution of Asian rice, and LOC_Os11g37050.1 could be missing or have large structural changes in ‘Silewah’. These assumptions are in agreement with the recessive inheritance of chilling tolerance for the Silewah allele. These considerations led to the conclusion that LOC_Os11g37050.1 is responsible for the *ctp-1* locus.

The annotation database showed that LOC_Os11g37050.1 encodes a protein similar to the NLR protein. NLR genes play a role for the recognition of pathogen-effector proteins, and most disease-resistance genes (*R* genes) in plants encode NLR proteins. NLR genes are responsible for immune responses against various pathogens, causing hypersensitive responses, including cell death processes (Dalio *et al.* 2020). Recently, several genes associated with chilling tolerance have been cloned in rice (Liu *et al.* 2013, 2018; Ma *et al.* 2015; Zhang *et al.* 2017; Zhao *et al.* 2017; Xiao *et al.* 2018; Mao *et al.* 2019; Zheng *et al.* 2022; Li *et al.* 2023). Those genes are involved in various physiological processes, such as hormonal signalling including jasmonate, abscisic acid and brassinosteroid signalling, cell wall metabolism and repairing DNA damage; however, no NLR genes have been reported to be involved in natural variation for chilling tolerance in rice.

A number of NLR genes in the plant genome that act as *R* genes are diversified in their function at the inter- and intra-species levels to meet the challenges of various biotic factors (Leister 2004). Comparison of genome sequences among species or subspecies demonstrated large genetic variation, including presence/absence and copy number polymorphisms of NLR genes, accompanied by changes in chromosomal structure (Leister 2004; Luo *et al.* 2012). LOC_Os11g37050.1, showed 99 % identity with LOC_Os11g38520.1 in duplicated chromosomal segment (Fig. 4 and Supporting Information—Fig. S3). LOC_Os11g38520.1 was reported as one of the five *Pb1* (panicle blast resistant gene 1) family genes in the ‘Nipponbare’ reference sequence (Hayashi *et al.* 2010). The *Pb1* gene was also suggested to be generated by tandem duplication of 60 kbp segments on the long arm of chromosome 11, resulting in the acquisition of a novel promoter sequence upstream of the coding sequence (Hayashi *et al.* 2010). Similar to *Pb1* family genes, such complex polymorphisms are often found in the rice genome, especially on chromosome 11 (The Rice Chromosomes 11 and 12 Sequencing Consortia 2005; Mizuno *et al.* 2020).

Differentiation of the *R* gene between populations or species might accidentally cause a maladaptive phenotype, resulting in a reduction in fitness of individuals. Hybrid incompatibility, such as hybrid necrosis, is a well-known example in which NLR genes with other interacting genes (differentiated among populations and coexisted by hybridization) cause necrotic phenotype (Calvo-Baltanas *et al.* 2020; Matsubara 2020; Wan *et al.* 2021). Our results suggest that the dominant (functional) allele might induce an autoimmune response by exposure to low temperatures, resulting in a chilling-susceptible phenotype. In contrast, the recessive (loss-of-function) allele might not induce an autoimmune response and show a tolerant phenotype. This remains to be examined through complementation tests or knockout of the candidate gene by genome editing, which may provide a better understanding of the molecular mechanisms involving the *ctp-1* gene in rice.

### Acclimation has an important role for adaptation in chilling stress environments in rice

Acclimation is a phenomenon that induces specific physiological and developmental processes prior to severe stress (Levitt 1980) and is known to increase freezing tolerance in temperate regions during autumn and the beginning of winter, so that these plants can survive severe winters (Thomashow 1999). Therefore, the ability of plants to acclimate is a key player in freezing tolerance; however, the roles of acclimation for chilling tolerance in plants from tropical–temperate regions are still unclear. In rice, few reports have indicated enhancement of chilling tolerance after acclimation treatments compared to non-acclimated plants (Baruah *et al.* 2011; Mao and Chen 2012; Xie *et al.* 2012). The present study demonstrated the nature of the acclimation response in rice and verified whether the *ctp-1* locus is involved in regulation of the acclimation response. Overlapping genetic mechanisms between acclimated and non-acclimated (intrinsic) tolerance have been suggested for freezing tolerance in Arabidopsis (Zhen and Ungerer 2008; Zuther *et al.* 2012). In contrast, intrinsic tolerance and acclimation ability were not correlated in the segregating population of potatoes (Stone *et al.* 1993). The present results clearly demonstrate that genes for acclimation response should be different from the *ctp-1* locus responsible for non-acclimated or intrinsic chilling tolerance because the genotype with chilling susceptible allele (*Ctp-1*^W107^ and *Ctp-1*^HY^) could respond to acclimation and enhance tolerance to a similar level of the tolerant allele (*ctp-1*^A58^).

The plumule of rice was observed to be fully acclimated to a wide range of temperatures (8 °C–16 °C). Acclimation in wheat, barley and rye also occurs at a wider range of temperatures, whereas the degree of freezing tolerance varies depending on the interaction between acclimation temperature, acclimation duration and genotype (Fowler 2008). Furthermore, the present results demonstrate that short-term exposure (12 h) to mild stress (8 °C) could rapidly suppress the susceptive response. A rapid acclimation response within 1 day has also been reported for freezing tolerance in Arabidopsis (Oono *et al.* 2006; Byun *et al.* 2014; Miki *et al.* 2019). Notably, Kidokoro *et al.* (2017) found that Arabidopsis plants respond differentially to rapid and gradual temperature decreases, which triggers different cold-responsive pathways depending on the light conditions. The chilling susceptible phenotype by the *Ctp-1* allele manifested with a rapid temperature shift from 30 °C to 0.5 °C, but not in a gradual shift by acclimation treatment, suggesting that rice plants might change their genetic pathways by responding to the pattern of temperature shift. Further detailed investigation is needed for physiological aspects of acclimation response in rice; however, acclimation response was considered possible to occur in natural environments where rice plants have been cultivated since temperature tends to gradually decrease in seasonal changes and fluctuates during the day.

In most studies, genetic analysis and screening for chilling-tolerant varieties have been conducted under constant, severe, low-temperature conditions for a few days without acclimation treatment, including our experiment (Baruah *et al.* 2009). Such artificial stress rarely occurs in natural environments where rice is cultivated. To date, only a few studies have examined the variation and genetics of acclimation ability in rice (Baruah *et al.* 2011; Mao and Chen 2012; Xie *et al.* 2012). Our previous results suggested that the wild rice strain W107 showed no or lower acclimation ability, as shown in strains of *indica*, which are mainly distributed in low latitudes and tropical regions (Baruah *et al.* 2011). Furthermore, acclimation ability shows a latitudinal cline for genetic variation in wild rice strains, which implies the importance of adaptation to the natural environment (Baruah *et al.* 2011). Revelation of different genetic factors for intrinsic tolerance and acclimation ability in the present study suggests that the inclusion of selection for acclimation ability in breeding programs should be advantageous to enhance chilling tolerance. Further analysis of the *ctp-1* locus and its interaction with acclimation gene(s) should provide new insights into the understanding of the genetic mechanism for adaptation to a low-temperature environment in rice.

## Supporting Information

The following additional information is available in the online version of this article—

Figure S1. The genetic variation for chilling tolerance at the plumule stage among varieties and breeding lines in Hokkaido.

Figure S2. PCR amplification in the candidate region of the *ctp-1* locus.

Figure S3. Comparison of DNA sequences of candidate genes for the *ctp-1* locus among A58 (LC783427), W107 (LC783428) and ‘Nipponbare’, and LOC_Os11g38520.1.

Figure S4. Comparison of amino acid sequences of candidate gene for the *ctp-1* locus among A58, W107 and ‘Nipponbare’ (A) and between W107 and ‘Nipponbare’ (B).

Figure S5. Chilling tolerance of J501 under different chilling temperature conditions at the plumule (A and B) and seedling stage (C and D).

Table S1. Primers of markers for fine mapping.

Table S2. Primers for sequencing.

plad075_suppl_Supplementary_Tables_S1

plad075_suppl_Supplementary_Tables_S2

plad075_suppl_Supplementary_Figures_S2

plad075_suppl_Supplementary_Figures_S1

plad075_suppl_Supplementary_Figures_S3

plad075_suppl_Supplementary_Figures_S4

plad075_suppl_Supplementary_Figures_S5

## Data Availability

The data generated during this work are included within the paper and its Supplementary Information files. The sequences of the candidate gene (LOC_Os11g37050.1) of A58 and W107 were deposited in DDBJ (accession numbers LC783427 and LC783428).
