# Supplementary material for: Genetics of chilling response at early growth stage in rice: a recessive gene for tolerance and importance of acclimation
Source: AoB Plants. 2023 Nov 8;15(6):plad075. doi: 10.1093/aobpla/plad075 (PMC10676198; doi:10.1093/aobpla/plad075)
Supplement: plad075_suppl_Supplementary_Tables_S1 [file plad075_suppl_supplementary_tables_s1.pdf]

Table S1. Primers of markers for fine mapping

	Primer L (5'-3')	Primer R (5'-3')
J03	TGCATCGATACAATCTACGG	TATGGCCGTTTCCTCTTCAC
P054	GCCTGAGTGGAACGCTAGTT	TCAGAATACGAAAGCTGTAAGG
C189	TAA GTT CTA CAT CGA CCC CA	CAC ATG AAG AGC TGG AAA CG
K21	GTAGTGTGTGGTGCTGACC	GGGACAGGAATAGAACCGAAC
AC137	GAGGACAGGCTGTCGTCATT	ATGTGCCAGAGGAATGGTTT
F10	CATCGATCCGTATGGGTTCT	AAAAATTACCCATGCGTTTAACT

All primers were reported in Baruah et al. (2009), except C189 (https://rgp.dna.affrc.go.jp/E/publicdata/caps/chr11.html).
