# Supplementary material for: Genetics of chilling response at early growth stage in rice: a recessive gene for tolerance and importance of acclimation
Source: AoB Plants. 2023 Nov 8;15(6):plad075. doi: 10.1093/aobpla/plad075 (PMC10676198; doi:10.1093/aobpla/plad075)
Supplement: plad075_suppl_Supplementary_Tables_S2 [file plad075_suppl_supplementary_tables_s2.pdf]

Table S2. Primers for sequencing

Primer name	Sequence (5’-3’)	Note
2011Long-L1	CAGCAGGGAGCATATGGATT	Sequencing
2011Long-R1	AACTGGATCGCCTACCACAC	Sequencing
2011.2nd-L1	GCAGACTGTGATGTGGAGGA	Sequencing
2011.2nd-R1	GAGCTCCCGCAAGATGTAGT	Sequencing
2011.2nd-L2	CCCTTCTCTGGACGACTACG	Sequencing
2011del-L1	GCCGAGCAAGCTACAAAGAC	Detection for deletion
2011del-R1	GCCTCTTCGTTTTGATCCAG	Detection for deletion
2011.2nd-L4	GAGGTATGGCGTTGAGATCC	Sequencing
2011.2nd-R2	CCAAGATGCCACGAAAAATC	Sequencing
2011.2nd-L3	GTCTTTGAGCCCCCTTCTCT	Sequencing
NcpsE L	TTTCAATACAATCCAGAAAATGCT	Sequencing
NcpsE R	AGTATCGAATGAGACATTTCTATTTTT	Sequencing
S1L	CACCATCCTGAGTTTCTTGAGAGTC	Sequencing
S1R	CACCATTTTAGCTCAATCAGAGGCC	Sequencing
S2L	AGATGCATTGATCTTCACCACAGGGA	Sequencing
S2R	TTGGCAAGTTTCTTATTACCACCTTC	Sequencing
up-1kb-L	GATACGGAAATACGAGATCATGC	Detection for SNP (marker SNPa)
up-1kb-R	TCCGATAAACGGAATTTACCTCT	Detection for SNP (marker SNPa)
dup.flank.r-L2	GGATCGCTAGAACGTGGAAC	Detection for SNP (marker SNPb)
dup.flank.r-R2	GGCACATCATCCATCAAGAA	Detection for SNP (marker SNPb)
