# Supplementary material for: Genetics of chilling response at early growth stage in rice: a recessive gene for tolerance and importance of acclimation
Source: AoB Plants. 2023 Nov 8;15(6):plad075. doi: 10.1093/aobpla/plad075 (PMC10676198; doi:10.1093/aobpla/plad075)
Supplement: plad075_suppl_Supplementary_Figures_S2 [file plad075_suppl_supplementary_figures_s2.pdf]

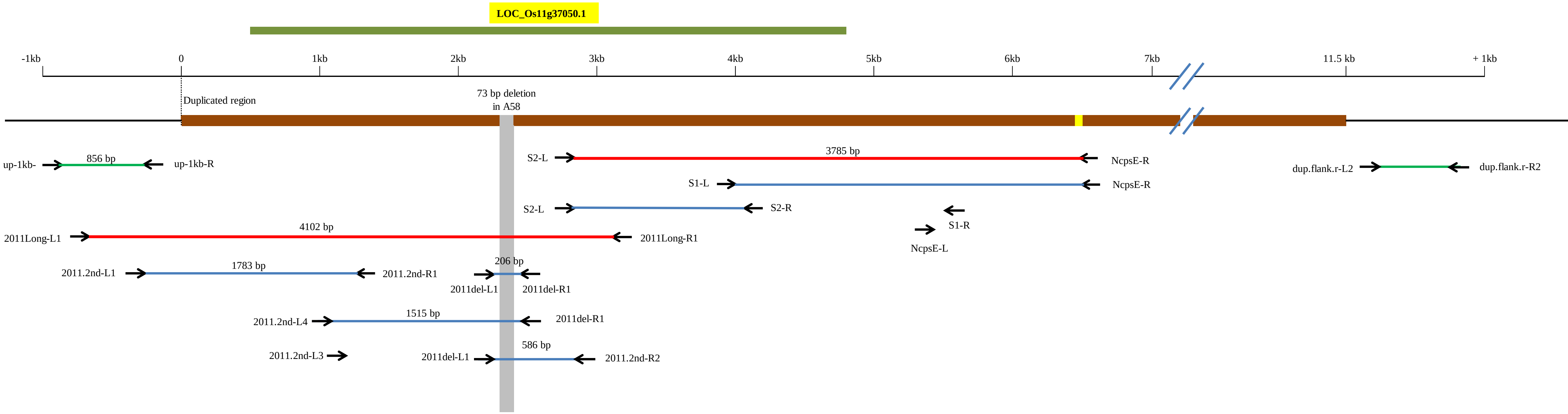


**Fig. S2**. PCR amplification in the candidate region of the *ctp-1* locus.

1st PCR    2nd PCR    For SNP genotyping
