# Supplementary material for: Genetics of chilling response at early growth stage in rice: a recessive gene for tolerance and importance of acclimation
Source: AoB Plants. 2023 Nov 8;15(6):plad075. doi: 10.1093/aobpla/plad075 (PMC10676198; doi:10.1093/aobpla/plad075)
Supplement: plad075_suppl_Supplementary_Figures_S1 [file plad075_suppl_supplementary_figures_s1.pdf]

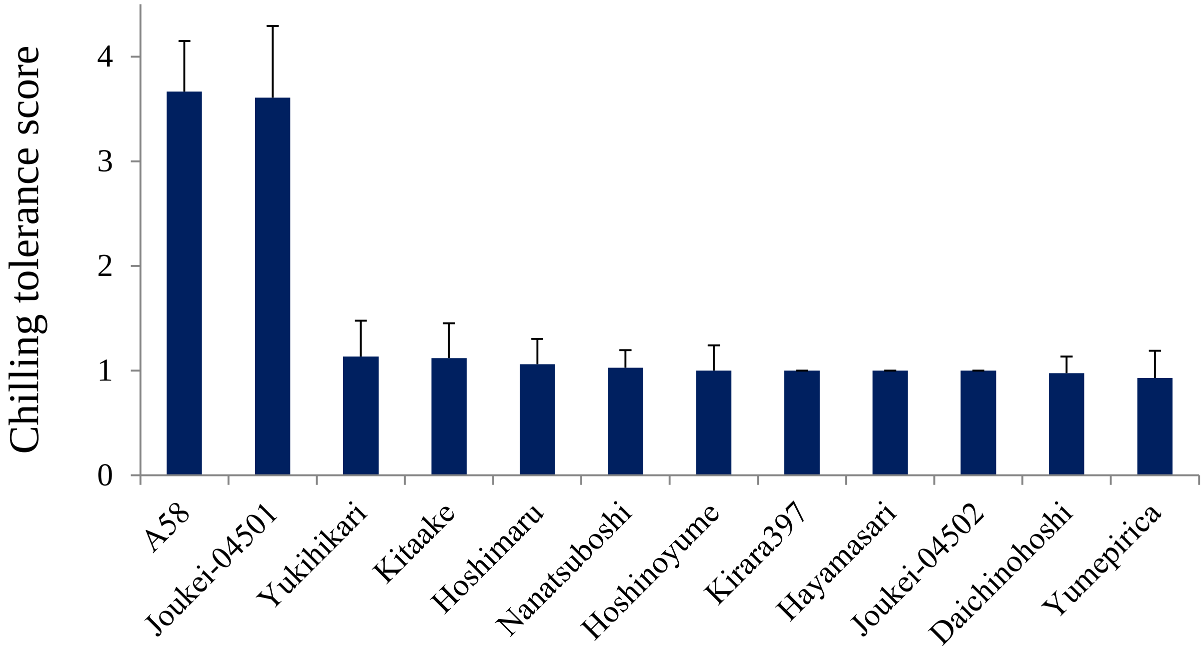

Fig. S1. The genetic variation of chilling tolerance at the plumule stage in Hokkaido varieties and breeding lines. Chilling tolerance score was evaluated as shown in Fig, 1A.
