# Supplementary material for: Genetics of chilling response at early growth stage in rice: a recessive gene for tolerance and importance of acclimation
Source: AoB Plants. 2023 Nov 8;15(6):plad075. doi: 10.1093/aobpla/plad075 (PMC10676198; doi:10.1093/aobpla/plad075)
Supplement: plad075_suppl_Supplementary_Figures_S3 [file plad075_suppl_supplementary_figures_s3.pdf]

Fig. S3. Comparison of DNA sequences of candidate genes for the *ctp-1* locus among A58 (LC783427), W107 (LC783428) and 'Nipponbare', and LOC_Os11g38520.1. DNA sequence of 'Hoshinoyume' (HY) is the same as 'Nipponbare.'

```
A58                   ATGACCGAGCTCGCGGCTGGCGCCGTGAGCTCACTGCTGGTGGTCATCCGCAACGAGGCT 60
W107                  ATGACCGAGCTCGCGGCTGGCGCCGTGAGCTCACTGCTGGTGGTCATCCGCAACGAGGCT 60
Nipponbare            ATGACCGAGCTCGCGGCTGGCGCCGTGAGCTCACTGCTGGTGGTCATCCGCAACGAGGCT 60
LOC_Os11g38520.1      ATGACCGAGCTCGCGGCTGGCGCCGTGAGCTCACTGCTGGTGGTCATCCGCAACGAGGCT 60
                      ************************************************************

A58                   GTGCTGCTTGGCGGCGTCCGGGACGATGTGCAGTTCATCAAGGAGGAGATGGAGAGCATG 120
W107                  GTGCTGCTTGGCGGCGTCCGGGACGATGTGCAGTTCATCAAGGAGGAGATGGAGAGCATG 120
Nipponbare            GTGCTGCTTGGCGGCGTCCGGGACGATGTGCAGTTCATCAAGGAGGAGATGGAGAGCATG 120
LOC_Os11g38520.1      GTGCTGCTTGGCGGCGTCCGGGACGATGTGCAGTTCATCAAGGAGGAGATGGAGAGCATG 120
                      ************************************************************

A58                   AACAGCTTCCTAGGGCACCTAGCCAGGAGCGCGCCGCAGGGTGGAGAGCACGACGAGCAG 180
W107                  AACAGCTTCCTGGGGCACCTAGCCAGGAGCGCGCCGCAGGGTGGAGAGCACGACGAGCAG 180
Nipponbare            AACAGCTTCCTAGGGCACCTAGCCAGGAGCGCGCCGCAGGGTGGAGAGCACGACGAGCAG 180
LOC_Os11g38520.1      AACAGCTTCCTGGGGCACCTAGCCAGGAGCGCGCCGCAGGGTGGAGAGCACGACGAGCAG 180
                      *********** ************************************************

A58                   GTGCGCACCTGGATGAACCAGGTGCGCCTGCTCGCGCAGGACTGCAACAACTGCATCGAC 240
W107                  GTGCGCACCTGGATGAACCAGGTGCGCCTGCTCGCGCAGGACTGCAACAACTGCATCGAC 240
Nipponbare            GTGCGCACCTGGATGAACCAGGTGCGCCTGCTCGCGCAGGACTGCAACAACTGCATCGAC 240
LOC_Os11g38520.1      GTGCGCACCTGGATGAACCAGGTGCGCCTGCTCGCGCAGGACTGCAACAACTGCATCGAC 240
                      ************************************************************

A58                   CTCTACCTCTACAGCGGGAACCCGGAGATCCACCGTGCCAAAGGTAGACTCCGGCGACAC 300
W107                  CTCTACCTCTACAGCGGGAACCCGGAGATCCACCGTACCAAAGGTAGACTCCGGCGACAC 300
Nipponbare            CTCTACCTCTACAGCGGGAACCCGGAGATCCACCGTGCCAAAGGTAGACTCCGGCGACAC 300
LOC_Os11g38520.1      CTCTACCTCTACAGCGGGAACCCGGAGATCCACCGTACCAAAGGTAGACTCCGGCGACAC 300
                      ************************************ ***********************

A58                   CTCTGGTGGGTCTACTGGTCCCTGCGCAAGATGGTCGCGCAGCACCGAGCGGCCATCCAG 360
W107                  CTCTGGTGGGTCTACTGGTCCCTGCGCAAGATGGTCGCGCAGCACCGAGCGGCCATCCAA 360
Nipponbare            CTCTGGTGGGTCTACTGGTCCCTGCGCAAGATGGTCGCGCAGCACCGAGCGGCCATCCAG 360
LOC_Os11g38520.1      CTCTGGTGGGTCTACTGGTCCCTGCGCAAGATGGTCGCGCAGCACCGAGCGGCCATCCAA 360
                      ***********************************************************

A58                   CTCCGCCAGCTCAAGGACCGGGCGCGGGACGTCGGCGAGCGACGACTGAGGTATGGCGTT 420
W107                  CTCCGCCAGCTCAAGGACCGGGCGCGGGACGTCGGCGAGCGACGACTGAGGTATGGCGTT 420
Nipponbare            CTCCGCCAGCTCAAGGACCGGGCGCGGGACGTCGGCGAGCGACGACTGAGGTATGGCGTT 420
LOC_Os11g38520.1      CTCCGCCAGCTCAAGGACCGGGCGCGGGACGTCGGCAAGCGACGACTGAGGTATGGCGTT 420
                      ************************************ ***********************

A58                   GAGATCCCAGCCACGACGAAGGCGGCCGCACCTGATGCAACAGGTGGCTATGCTGCTGGG 480
W107                  GAGATCCCAGCCACGACGAAGGCGGCCGCACCTGATGCAACAGGTGGCTATGTTGCTGGG 480
Nipponbare            GAGATCCCAGCCACGACGAAGGCGGCCGCACCTGATGCAACAGGTGGCTATGCTGCTGGG 480
LOC_Os11g38520.1      GAGATCCCAGCCACGACGAAGGCGGCCGCACCTGATGCAACAGGTGGCTATGTTGCTGAG 480
                      **************************************************** ***** *
```

```
A58                 GACGATGAAGAAGAAGATGAAGATGACCGCGAAGGACAATTTGCTGTGGCAACACCAACG 540
W107                GACGATGAAGAAGAAGATGAAGATGACCGCGAAGGACAATTTGCTGTGGCAACACCAACG 540
Nipponbare          GACGATGAAGAAGAAGATGAAGATGACCGCGAAGGACAATTTGCTGTGGCAACACCAACG 540
LOC_Os11g38520.1    GACGATGAAGAAGAAGATGAAGATGACCGCGAAGGACAATTTGCTGTGGCAACACCAACG 540
                    ************************************************************

A58                 TTGGCCCATCATTCTGCTCGATGGCCCGTCTTTGAGCCCCCTTCTCTGGACGACTACGTG 600
W107                TTGGCCCATCATTCTGCTCGATGGCCCGTCTTTGAGCCCCCTTCTCTGGACGACTACGTG 600
Nipponbare          TTGGCCCATCATTCTGCTCGATGGCCCGTCTTTGAGCCCCCTTCTCTGGACGACTACGTG 600
LOC_Os11g38520.1    TTGGCCCATCATTCTGCTCGATGGCCCGTCTTTGAGCCCCCTTCTCTGGACGACTACGTG 600
                    ************************************************************

A58                 AAGGCCAAGTTATTGGAGTGGATTGAGGGAGTTCCTGGAAACGCCATCGTGACGTTGTCC 660
W107                AAGGCAAAGTTATTGGAGTGGATTGGGGGAGTTCCTGGAAACGCCATCGTGACGTTGTCC 660
Nipponbare          AAGGCCAAGTTATTGGAGTGGATTGAGGGAGTTCCTGGAAACGCCATCGTGACGTTGTCC 660
LOC_Os11g38520.1    GAGGCGAAGTTATTGGAGTGGATTGGGGGAGTTCCTGGAAACGCCATCGTGACGTTGTCC 660
                     **** ******************* **********************************

A58                 ATCGCCATTGTAGCACCAGATGCAGATAATAAGGAAGTCCTTGCTATTGCACATGAAACT 720
W107                ATCGCCATTGTAGCACCAGATGCAGATAATAAGGAAGTCCTTGCTATTGCACATGAAACT 720
Nipponbare          ATCGCCATTGTAGCACCAGATGCAGATAATAAGGAAGTCCTTGCTATTGCACATGAAACT 720
LOC_Os11g38520.1    ATCGCCATTGTAGCACCAGATGCAGATAATAAGGAAGTCCTTGCTATTGCACATGAAACT 720
                    ************************************************************

A58                 TTGGTTGCTCCAGACTACTACTACCGTCGCAGTATCATGGTCAACGTCCCGGCGGTGCAC 780
W107                TTGGTTGCTCCAAACTACTACTACCGTCGCAGTATCATGGTCAACGTCCCGGCGGTGCAC 780
Nipponbare          TTGGTTGCTCCAGACTACTACTACCGTCGCAGTATCATGGTCAACGTCCCGGCGGTGCAC 780
LOC_Os11g38520.1    TTGGTTGCTCCAAACTACTACTACCGTCGCAGTATCATGGTCAACGTCCCGGCGGTGCAC 780
                    ************ ***********************************************

A58                 CTTGATTTTTTACCGCTTCGACCCAAGGAGGTCCTCTACTACATCTTGCGGGAGCTCGAG 840
W107                CTTGATTTTTTACCGCTTCGACCCAAGGAGGTCCTCTACTACATCTTGCGGGAGCTCGAG 840
Nipponbare          CTTGATTTTTTACCGCTTCGACCCAAGGAGGTCCTCTACTACATCTTGCGGGAGCTCGAG 840
LOC_Os11g38520.1    CTTGATGTTTTACCGCTTCGACCCAAGGAGGTCCTCTACTACATCTTGCGGGAGCTCGAG 840
                    ****** *****************************************************

A58                 CGGGAAGAAGCGGCCGGATCGCAGAAGCAGCCCACAGATCAAGGTGAATGGGAGGAGGAG 900
W107                CGGGAAGAAGCGGCCGGATCGCAGAAGCAGCCCACAGATCAAGGTGAATGGGAGGAGGAG 900
Nipponbare          CGGGAAGAAGCGGCCGGATCGCAGAAGCAGCCCACAGATCAAGGTGAATGGGAGGAGGAG 900
LOC_Os11g38520.1    CGGGAAGAAGCGGCCGGATCGCAGAAGCAGCCCACAGATCAAGGTGAATGGGAGGAGGAG 900
                    ************************************************************

A58                 GATCCTGACCCTTGGCAAGATTACTACAAAAAATGTGGCATTTACCGTAGCAAAAAAGGT 960
W107                GATCCTGACCCTTGGCAAGATTACTACAAAAAATGTGGCATTTACCGTAGCAAAAAAAGT 960
Nipponbare          GATCCTGACCCTTGGCAAGATTACTACAAAAAATGTGGCATTTACCGTAGCAAAAAAGGT 960
LOC_Os11g38520.1    GATCCTGACCCTTGGCAAGATTACTACAAAAAATGTGGCATTTACCGTAGCAAAAAAAGT 960
                    ********************************************************* **

A58                 GTGCTCGATAAAATCAAAAGGAATATAAAAAAGATGAATATTTACGAAAAGCTTGATAAG 1020
W107                GTGCTCGGTAAAATCAAAAGGAATATAAAAAAGATGAATATTTACGAAAAGCTTGATAAG 1020
Nipponbare          GTGCTCGATAAAATCAAAAGGAATATAAAAAAGATGAATATTTACGAAAAGCTTGATAAG 1020
LOC_Os11g38520.1    GTGCTCGGTAAAATCAAAAGGAATATAAAAAAGATGAATATTTACGAAAAGCTTGATAAG 1020
                    ******* ****************************************************
```

```
A58              ATCAAGAGCGACATTCGAGAAGGACAACACAAGAGCGGCAAGCTGTTGCTGCTTAAGCTA 1080
W107             ATCAAGAGCGACATTCGAGAAGGACAACACAAGAGCGACAAGCTGTTGCTGCTTCAGCTA 1080
Nipponbare       ATCAAGAGCGACATTCGAGAAGGACAACACAAGAGCGGCAAGCTGTTGCTGCTTAAGCTA 1080
LOC_Os11g38520.1 ATCAAGAGCGACATTCGAGAAGGACAACACAAGAGCAACAAGCTGTTGCTGCTTCAGCTA 1080
                 ************************************  **************** *****

A58              CAAGAGAAAGGTGCGGATCAGGTGGACCTACATGTACTCCTTCAGCTGTTGGTGCTCCAG 1140
W107             CAAAAGAAAGGTGCGGATCAGGTGGACCTACATGTACTCCTTCAGCTGTTGGTGCTCCAG 1140
Nipponbare       CAAGAGAAAGGTGCGGATCAGGTGGACCTACATGTACTCCTTCAGCTGTTGGTGCTCCAG 1140
LOC_Os11g38520.1 CAAAAGAAAGGTGCGGATCAGGTGGACCTACATGTACTCCTTCAGCTGTTGGTGCTCCAG 1140
                 *** ********************************************************

A58              TCTCAGCAAGACCAAGCGAAGAACAAAGCAGTAGACACCCATAAATTACCAGAGTGGAAC 1200
W107             TCTCAGCAAGACCAAGCGAAGAACAAAGCAGTAGACACCCATAAATTACCAGAGTGGAAC 1200
Nipponbare       TCTCAGCAAGACCAAGCGAAGAACAAAGCAGTAGACACCCATAAATTACCAGAGTGGAAC 1200
LOC_Os11g38520.1 TCTCAGCAAGACCAAGCGAAGAACAAAGCAGTAGACACCCATAAATTACCAGAGTGGAAC 1200
                 ************************************************************

A58              GACAACCTCATAGAAAAATTAGCCATGAGGCTGAAGGATCATATGGAAGCAGATGAAAAG 1260
W107             GACAACCTCATAGAAAAATTAGCCATGAGGCTGAAGGATCATATGGAAGCAGATGAAAAG 1260
Nipponbare       GACAACCTCATAGAAAAATTAGCCATGAGGCTGAAGGATCATATGGAAGCAGATGAAAAG 1260
LOC_Os11g38520.1 GACAACCTCATAGAAAAATTAGCCATGAGGCTGAAGGATCATATGGAAGCAGATGAAAAG 1260
                 ************************************************************

A58              ACCAAGAAGCTTAATGAGCAAACTGGAGTAGAAGAAGAAACAGCAGTCAGACAAGGCGGA 1320
W107             ACCAAGAAGCTTAATGAGCAAACTGGAGTAGAAGAAGAAACAGCAGTCAGACAAGGCGGA 1320
Nipponbare       ACCAAGAAGCTTAATGAGCAAACTGGAGTAGAAGAAGAAACAGCAGTCAGACAAGGCGGA 1320
LOC_Os11g38520.1 ACCAAGAAGCTTAATGAGCAAACTGGAGTAGAAGAAGAAACAGCAGTCAGACAAGGCGGA 1320
                 ************************************************************

A58              GGAGGAGAGAGGAAGGAGGATGAGAAAGACGAAAGAGGAGACGGAGAAGAAGAGGGGAAG 1380
W107             GGAGGAGAGAGGGAGGAGGATGAGAAAGACGAAAGAGGAGACGGAGAAGAAGAGGGGAAG 1380
Nipponbare       GGAGGAGAGAGGAAGGAGGATGAGAAAGACGAAAGAGGAGACGGAGAAGAAGAGGGGAAG 1380
LOC_Os11g38520.1 GGAGGAGAGAGGGAGGAGGATGAGAAAGACGAAAGAGGAGACGGAGAAGAAGAGGGGAAG 1380
                 ************ ***********************************************

A58              GAGGAGAGGAGGGACATGGAGAAAGG---AGAAGAGAGGAAGGAGCAGCAGCAGGAGGAG 1437
W107             GAGGAGAGGAGGGACATGGAGAAAGGAGGAGAAGAGAGGAAGGAGCAGCAGCAGGAGGAG 1440
Nipponbare       GAGGAGAGGAGGGACATGGAGAAAGG---AGAAGAGAGGAAGGAGCAGCAGCAGGAGGAG 1437
LOC_Os11g38520.1 GAGGAGAGGAGGGACATGGAGAAAGGAGGAGAAGAGAGGAAGGAGCAGCAGCAGGAGGAG 1440
                 **************************   *******************************

A58              CAGGAGAAAGAAGGGAGGAAGGAGGAACAGAACGAGGTAAGAAAGGAGACAGAAGGAAGA 1497
W107             CAGGAGAAAGAAGGGAGGAAGGAGGAACAGAACGAGGTAAGAAAGGAGACAGAAGGAAGA 1500
Nipponbare       CAGGAGAAAGAAGGGAGGAAGGAGGAACAGAACGAGGTAAGAAAGGAGACAGAAGGAAGA 1497
LOC_Os11g38520.1 CAGGAGAAAGAAGGGAGGAAGGAGGAACAGAACGAGGTAAGAAAGGAGACAGAAGGAAGA 1500
                 ************************************************************

A58              AAAGAACAAGTAGCAGGAGAGGAGGAAGAAAAAGAAGACCATGATGCTGATAATGATGAA 1557
W107             AAAGAACAAGTAGCAGGAGAGGAGGAAGAAAAAGAAGACCATGATGCTGATAATGATGAA 1560
Nipponbare       AAAGAACAAGTAGCAGGAGAGGAGGAAGAAAAAGAAGACCATGATGCTGATAATGATGAA 1557
LOC_Os11g38520.1 AAAGAACAAGTAGCAGGAGAGGAGGAAGAAAAAGAAGACCATGATGCTGATAATGATGAA 1560
                 ************************************************************
```

```
A58                 GACAGCAATGATGATGATGATGATGATGAGGAGGAGGAGGAGGA------CGACGATGAT 1611
W107                GACAGCAATGATGATGATGATGATGAGGAGGAGGAGGAGGAGGAGGAGGACGACAATGAT 1620
Nipponbare          GACAGCAATGATGATGATGATGATGAGGAGGAGGAGGAGGAGGA------CGACGATGAT 1611
LOC_Os11g38520.1    GACAGCAATGATGATGATGATGATGAGGAGGAGGAGGAGGAGGACGA---CGACGATGAT 1617
                    ************************** *****************      **** *****

A58                 GAAGAAGAACCAATTCATCTTCATGAAGATCAATATGAACAAATCCTACGAGAAGTGTTC 1671
W107                GAAGAAGAACCAATTCATCTTCATGAAGATCAATATGAACAAATCCTACGAGAAGTGTTC 1680
Nipponbare          GAAGAAGAACCAATTCATCTTCATGAAGATCAATATGAACAAATCCTACGAGAAGTGTTC 1671
LOC_Os11g38520.1    GAAGAAGAACCAATTCATCTTCATGAAGATCAATATGAACAAATCCTACGAGAAGTGTTC 1677
                    ************************************************************

A58                 ACAAAGAACGCCAGCAGCAAGGCCCAGGAGCAAGATAAATTGGTGGCCGAGCAAGCTACA 1731
W107                ACAAAGAACGCCAGCAGCAAGGCCCAGGAGCAAGATAAATTGGTGGCCGAGCAAGCTACA 1740
Nipponbare          ACAAAGAACGCCAGCAGCAAGGCCCAGGAGCAAGATAAATTGGTGGCCGAGCAAGCTACA 1731
LOC_Os11g38520.1    ACAAAGAACGCCAGCAGCAAGGCCCAGGAGCAAGATAAATTGGTGGCCGAGCAAGCTACA 1737
                    ************************************************************

A58                 AAGACTGCAGCCACTACATTGGATGAGGAACGAATCAAACAAATGATCTA---------- 1784
W107                AAGACTGCAGCCACTACATTGGATGAGGAACGAATCAAACAAATGATCAACGAAGCAAAA 1800
Nipponbare          AAGACTGCAGCCACTACATTGGATGAGGAACGAATCAAACAAATGATCAACGAAGCAAAA 1791
LOC_Os11g38520.1    AAGACTGCAGCCACTACATTGGATGAGGAACGAATCAAACAAATGATCAACGAAGCAAAA 1797
                    ************************************************ *

A58                 ------------------------------------------------------------
W107                CAAGATGTCTTAAGGGAGCTGCGGGGACGCGAAACTGATAAGAATCAAGCAACAGGTGAA 1860
Nipponbare          CAAGATGTCTTAAGGGAGCTGCGGGGACGCGAAACTGATAAGAATCAAGCAACAGGTGAA 1851
LOC_Os11g38520.1    CAAGATGTCTTAAGGGAGCTGCGGGGACGCGAAACTGATAAGAATCAAGCAACAGGTGAA 1857


A58                 ---TAGGTTCCACCTGATAAGAATCAAGCAACAGGTCAACATGCAGTTGTTCTGGATCAA 1838
W107                CCTGATGTTCCACCTGATAAGAATCAAGCAACAGGTCAACATGCAGTTGTTCTGGATCAA 1920
Nipponbare          CCTGATGTTCCACCTGATAAGAATCAAGCAACAGGTCAACATGCAGTTGTTCTGGATCAA 1911
LOC_Os11g38520.1    CCTGATGTTCCACCTGATAAGAATCAAGCAACAGGTCAACATGCAGTTGTTCTGGATCAA 1917
                        * ******************************************************

A58                 AACGAAGAGGCTTATTTTGAAGAAGTAGAGCAGAAGATTGAAGAAATCAAGCAGGAACTT 1898
W107                AACGAAGAGGCTTATTTTGAAGAAGTAGAGCAGAAGATTGAAGAAATCAAGCAGGAACTT 1980
Nipponbare          AACGAAGAGGCTTATTTTGAAGAAGTAGAGCAGAAGATTGAAGAAATCAAGCAGGAACTT 1971
LOC_Os11g38520.1    AACGAAGAGGCTTATTTTGAAGAAGTAGAGCAGAAGATTGAAGAAATCAAGCAGGAACTT 1977
                    ************************************************************

A58                 AAAGAGCAGCTGAAGATCAAATGGATCGTGGACAAGATTAAACATCACTTACAGGATCAG 1958
W107                AAAGAGCAGCTGAAGATCAAATGGATCGTGGACAAGATTAAACATCACTTACAGGATCAG 2040
Nipponbare          AAAGAGCAGCTGAAGATCAAATGGATCGTGGACAAGATTAAACATCACTTACAGGATCAG 2031
LOC_Os11g38520.1    AAAGAGCAGCTGAAGATCAAATGGATCGTGGACAAGATTAAACATCACTTACAGGATCAG 2037
                    ************************************************************

A58                 TGCCCCCTCATTATCCTAAAATTTGATCAGATGATGGATGGATCCCGATGGGAGGAGATC 2018
W107                TGCCCCCTCATTATCCTAAAATTTGATCAGATGATGGATGGATCCCGATGGGAGGAGATC 2100
Nipponbare          TGCCCCCTCATTATCCTAAAATTTGATCAGATGATGGATGGATCCCGATGGGAGGAGATC 2091
LOC_Os11g38520.1    TGCCCCCTCATTATCCTAAAATTTGATCAGATGATGGATGGATCCCGATGGGAGGAGATC 2097
                    ************************************************************
```

```
A58                 AGAAAGGCTCTGAGCCTGTTAGAATTGAGTGCAGATGCATTGATCTTCACCACAGGGAGC 2078
W107                AGAAAGGCTCTGAGCCTGTTAGAATTGAGTGCCGATGCATTGATCTTCACCACAGGGAGC 2160
Nipponbare          AGAAAGGCTCTGAGCCTGTTAGAATTGAGTGCAGATGCATTGATCTTCACCACAGGGAGC 2151
LOC_Os11g38520.1    AGAAAGGCTCTGAGCCTGTTAGAATTGAGTGCCGATGCATTGATCTTCACCACAGGGAGC 2157
                    ******************************** ***************************

A58                 ACAGAACAGGCTAAAGGATATTGCTATCCACCACGAGAACCTATAGATCACTGTTCTCTT 2138
W107                ACAGAACAGGCTAAAGGATATTGCTATCCACCACGAGAACCTATAGATCACTGTTCTCTT 2220
Nipponbare          ACAGAACAGGCTAAAGGATATTGCTATCCACCACGAGAACCTATAGATCACTGTTCTCTT 2211
LOC_Os11g38520.1    ACAGAACAGGCTAAAGGATATTGCTATCCACCACGAGAACCTATAGATCACTGTTCTCTT 2217
                    ************************************************************

A58                 GTTGGTCTCTACTATTATACGGTGCTCAAGCTTACTAGCAAGCACAAGAATGAAGACAAC 2198
W107                GTTGGTCTCTACTATTATACGGTGCTCAAGCTTACTAGCAAGCACAAGAATGAAGACAAC 2280
Nipponbare          GTTGGTCTCTACTATTATACGGTGCTCAAGCTTACTAGCAAGCACAAGAATGAAGACAAC 2271
LOC_Os11g38520.1    GTTGGTCTCTACTATTATACGGTGCTCAAGCTTACTAGCAAGCACAAGAATGAAGACAAC 2277
                    ************************************************************

A58                 GATAACGCACAGATTTTTCGTGGCATCTTGGAGGAGTGTGAGGGGCATGAATTCTGCATG 2258
W107                GATAACGCACAGATTTTTCGTGGCATCTTGGAGGAGTGTGAGGGGCATGAATTCTGCATG 2340
Nipponbare          GATAACGCACAGATTTTTCGTGGCATCTTGGAGGAGTGTGAGGGGCATGAATTCTGCATG 2331
LOC_Os11g38520.1    GATAACGCACAGATTTTTCGTGGCATCTTGGAGGAGTGTGAGGGGCATGAATTCTGCATG 2337
                    ************************************************************

A58                 AAGATCTTCACTCATGCTGTGTATGCTAATCCCAAGAGGAGCAATGAAGAGTTAAGGAAG 2318
W107                AAGATCTTCACTCATGCTGTGTATGCTAATCCCAAGAGGAGCAATGAAGAGTTAAGGAAG 2400
Nipponbare          AAGATCTTCACTCATGCTGTGTATGCTAATCCCAAGAGGAGCAATGAAGAGTTAAGGAAG 2391
LOC_Os11g38520.1    AAGATCTTCACTCATGCTGTGTATGCTAATCCCAAGAGGAGCAATGAAGAGTTAAGGAAG 2397
                    ************************************************************

A58                 CTACACAGCACCCTGCAGTCTCCAAAAAAATCATTCGACACCATAGCTAAGAAGATGTTC 2378
W107                CTACACAGCACCCTGCAGTCTCCAAAAAAATCATTCGACACCATAGCTAAGAAGATGTTC 2460
Nipponbare          CTACACAGCACCCTGCAGTCTCCAAAAAAATCATTCGACACCATAGCTAAGAAGATGTTC 2451
LOC_Os11g38520.1    CTACACAGCACCCTGCAGTCTCCAAAAAAATCATTCGACACCATAGCTAAGAAGATGTTC 2457
                    ************************************************************

A58                 ATGTACTCTTACAATGATCTACCTAAAGAATACAAGTCATGCTTGCTGTACCTAGCTATC 2438
W107                ATGTACTCTTACAATGATCTACCTAAAGAATACAAGTCATGCTTGCTGTACCTAGCTATC 2520
Nipponbare          ATGTACTCTTACAATGATCTACCTAAAGAATACAAGTCATGCTTGCTGTACCTAGCTATC 2511
LOC_Os11g38520.1    ATGTACTCTTACAATGATCTACCTAAAGAATACAAGTCATGCTTGCTGTACCTAGCTATC 2517
                    ************************************************************

A58                 TTCCCCAAGGGACAGAAGATTAGGCGGTCAACCTTGATTGCAAGGTGGGTTGCAGAAGGG 2498
W107                TTCCCCAAGGGACAGAAGATTAGGCGGTCAACCTTGATTGCAAGGTGGGTTGCAGAAGGG 2580
Nipponbare          TTCCCCAAGGGACAGAAGATTAGGCGGTCAACCTTGATTGCAAGGTGGGTTGCAGAAGGG 2571
LOC_Os11g38520.1    TTCCCCAAGGGACAGAAGATTAGGCGGTCAACCTTGATTGCAAGGTGGGTTGCAGAAGGG 2577
                    ************************************************************

A58                 TTGACATTCAAGGAAGATTGGCCCAGCTCTGTGTATCAGGCAAATCGATGTTTTGATGCC 2558
W107                TTGACATTCAAGGAAGATTGGCCCAGCTCTGTGTATCAAGCAAATCGATGTTTTGATGCC 2640
Nipponbare          TTGACATTCAAGGAAGATTGGCCCAGCTCTGTGTATCAGGCAAATCGATGTTTTGATGCC 2631
LOC_Os11g38520.1    TTGACATTCAAGGAAGATTGGCCCAGCTCTGTGTATCAAGCAAATCGATGTTTTGATGCC 2637
                    ************************************** *********************
```

```
A58                 CTCATCCGTCGGTGGCTTGTTTATCCTGATGATATTAGTGCCACAGGAAAGATCAAGAGC 2618
W107                CTCATCCGTCGGTGGCTTGTTTATCCTGATGATATTAGTGCCACAGGAAAGATCAAGAGC 2700
Nipponbare          CTCATCCGTCGGTGGCTTGTTTATCCTGATGATATTAGTGCCACAGGAAAGATCAAGAGC 2691
LOC_Os11g38520.1    CTCATCCGTCGGTGGCTTGTTTATCCTGATGATATTAGTGCCACAGGAAAGATCAAGAGC 2697
                    ************************************************************

A58                 TGTGTGGTAGGCGATCCAGTTCATGGGTTCATTACCGCAATTGCCAGAAAACAACATATT 2678
W107                TGTGTGGTAGGCGATCCAGTTCATGGGTTCATTACCGCAATTGCCAGAAAACAACATATT 2760
Nipponbare          TGTGTGGTAGGCGATCCAGTTCATGGGTTCATTACCGCAATTGCCAGAAAACAACATATT 2751
LOC_Os11g38520.1    TGTGTGGTAGGCGATCCAGTTCATGGGTTCATTACCGCAATTGCCAGAAAACAACATATT 2757
                    ************************************************************

A58                 GTGGAGACACGCCTATCACATCACTTGGCTCGCCACTTCTCCATTTTCAATGATCTTCGA 2738
W107                GTGGAGACACGCCTATCTCATCACTTGGCTCGCCACTTCTCCATTTTCAATGATCTTCGA 2820
Nipponbare          GTGGAGACACGCCTATCACATCACTTGGCTCGCCACTTCTCCATTTTCAATGATCTTCGA 2811
LOC_Os11g38520.1    GTGGAGACACGCCTATCTCATCACTTGGCTCGCCACTTCTCCATTTTCAATGATCTTCGA 2817
                    ***************** ******************************************

A58                 CTCCGCAGCTCTGATAGAATTGGCACGTTCTTCCAAGGCCTCTCACGATCATCTCGAGTA 2798
W107                CTCCGCAGCTCTGATAGAATTGGCACGTTCTTCCAAGGCCTCTCACGATCATCTCGAGTA 2880
Nipponbare          CTCCGCAGCTCTGATAGAATTGGCACGTTCTTCCAAGGCCTCTCACGATCATCTCGAGTA 2871
LOC_Os11g38520.1    CTCCGCAGCTCTGATAGAATTGGCACGTTCTTCCAAGGCCTCTCACGATCATCTCGAGTA 2877
                    ************************************************************

A58                 TCCCTACTCAAGGTGCTAGATCTAGAAGGTTGTCAGTGCTTTGCTAGTAAGAATCAGCGG 2858
W107                TCCCTACTCAAGGTGCTAGATCTAGAAGGTTGTCAGTGCTTTGCTAGTAAGAATCAGCGG 2940
Nipponbare          TCCCTACTCAAGGTGCTAGATCTAGAAGGTTGTCAGTGCTTTGCTAGTAAGAATCAGCGG 2931
LOC_Os11g38520.1    TCCCTACTCAAGGTGCTAGATCTAGAAGGTTGTCAGTGCTTTGCTAGTAAGAATCAGCGG 2937
                    ************************************************************

A58                 TACCTCAAGGACATCTGCAACAAGATGTTACTGCTCAAATATCTGAGCCTAAAGGGAACA 2918
W107                TACCTCAAGGACATCTGCAACAAGATGTTACTGCTCAAATATCTGAGCCTAAAGGGAACA 3000
Nipponbare          TACCTCAAGGACATCTGCAACAAGATGTTACTGCTCAAATATCTGAGCCTAAAGGGAACA 2991
LOC_Os11g38520.1    TACCTCAAGGACATCTGCAACAAGATGTTACTGCTCAAATATCTGAGCCTAAAGGGAACA 2997
                    ************************************************************

A58                 GATATTACCCAGCTGCCCAGTGAAATCAACTGCCTCCGCGAGCTAGAGGTATTGGATATC 2978
W107                GATATTACCCAGCTGCCCAAGGAAATCAACTGCCTCCGCGAGCTAGAGGTATTGGATATC 3060
Nipponbare          GATATTACCCAGCTGCCCAGTGAAATCAACTGCCTCCGCGAGCTAGAGGTATTGGATATC 3051
LOC_Os11g38520.1    GATATTACCCAGCTGCCCAAGGAAATCAACTGCCTCCGCGAGCTAGAGGTATTGGATATC 3057
                    *******************  ***************************************

A58                 CGAGAAACCAAGGTGCCTGCAAATGCAACAGTAAATGTCCTGCTCTTGAAGCTGAAGCGT 3038
W107                CGAGAAACCAAGGTGCCTGCAAATGCAACAGTACATGTCCTGCTCTTGAAGCTGAAGCGT 3120
Nipponbare          CGAGAAACCAAGGTGCCTGCAAATGCAACAGTAAATGTCCTGCTCTTGAAGCTGAAGCGT 3111
LOC_Os11g38520.1    CGAGAAACCAAGGTGCCTGCAAATGCAACAGTACATGTCCTGCTCTTGAAGCTGAAGCGT 3117
                    ********************************* **************************

A58                 CTACTTGCTGGTGCTAGTCAGATTGATCCAACTCCAAGAAATTTTGTCACTAACGTCCGG 3098
W107                CTACTTGCTGGTGCTAGTCAGAATGATCCAACTCCAAGAAATTTTGTCACTAACGTCCGG 3180
Nipponbare          CTACTTGCTGGTGCTAGTCAGATTGATCCAACTCCAAGAAATTTTGTCACTAACGTCCGG 3171
LOC_Os11g38520.1    CTACTTGCTGGTGCTAGTCAGATTGATCCAACTCCAAGAAATTTTGTCACTAACGTCCGG 3177
                    ********************** *************************************
```

```
A58              ATTCCTTCCAGGATAGACAAGATGATAAACATAGAGGTACTATCTAATGTCAAGGCCCAG 3158
W107             ATTCCTTCCAGGATAGACAAGATGATAAACATAGAGGTACTATCTAATGTCAAGGCCCAG 3240
Nipponbare       ATTCCTTCCAGGATAGACAAGATGATAAACATAGAGGTACTATCTAATGTCAAGGCCCAG 3231
LOC_Os11g38520.1 ATTCCTTCCAGGATAGACAAGATGATAAACATAGAGGTACTATCTAATGTCAAGGCCCAG 3237
                 ************************************************************

A58              CACCATGATAATTTGGAAGATATTGGGAAGCTATGCCAGTTGAGGAAGCTAGGTGTGGTT 3218
W107             CACCATGATAATTTGGAAGATATTGGGAAGCTATGCCAGTTGAGGAAGCTAGTTGTGGTT 3300
Nipponbare       CACCATGATAATTTGGAAGATATTGGGAAGCTATGCCAGTTGAGGAAGCTAGGTGTGGTT 3291
LOC_Os11g38520.1 CAACATGATAATTTGGAAGATATTGGGAAGCTATGCCAGTTGAGGAAGCTAGGTGTGGTT 3297
                 ** ************************************************* *******

A58              ATTGATGGTAAGAAAAGTCACCTTGGGAGTTTGCTTAAAGCGATCAGTGACCTACATGCA 3278
W107             ATTGATGGTAAGAAAAGTCACCTTGGGAGTTTGCTTAAAGCGATCAGTGACCTACATGCA 3360
Nipponbare       ATTGATGGTAAGAAAAGTCACCTTGGGAGTTTGCTTAAAGCGATCAGTGACCTACATGCA 3351
LOC_Os11g38520.1 ATTGATGGTAAGAAAAGTCACCTTGGGAGTTTGCTTAAAGCGATCAGTGACCTACATGCA 3357
                 ************************************************************

A58              AGCCTCCGTTCTCTGTCAATCACTATTCCCACAACCACACTCGAGGTTACTCCTTCAAGC 3338
W107             AGCCTCCGTTCTCTGTCAATCACTATTCCCACAACCACACTCGAGGTTACTCCTTCAAGC 3420
Nipponbare       AGCCTCCGTTCTCTGTCAATCACTATTCCCACAACCACACTCGAGGTTACTCCTTCAAGC 3411
LOC_Os11g38520.1 AGCCTCCGTTCTCTGTCAATCACTATTCCCACAACCACACTCGAGGTTACTCCTTCAAGC 3417
                 ************************************************************

A58              CCAGAGTTACAAGATATTGCATCTCGCCTAAAACACCATCCTGAGTTTCTTGAGAGTCTA 3398
W107             CCAGAGTTACAAGATATTGCATCTCGCCTAAAACACCATCCTGAGTTTCTTGAGAGTCTA 3480
Nipponbare       CCAGAGTTACAAGATATTGCATCTCGCCTAAAACACCATCCTGAGTTTCTTGAGAGTCTA 3471
LOC_Os11g38520.1 CCAGAGTTACAAGATATTGCATCTCGCCTAAAAGACCATCCTGAGTTTCTTGAGAGTCTA 3477
                 ********************************* **************************

A58              AGCATCAGTGGAGCCAAGCATCTTTTTCCATTGTTGACCGAAGGTGGTAATAAGAAACTT 3458
W107             AGCATCAGTGGAGCCAAGCATCTTTTTCCATTGTTGACCGAAGGTGGTAATAAGAAACTT 3540
Nipponbare       AGCATCAGTGGAGCCAAGCATCTTTTTCCATTGTTGACCGAAGGTGGTAATAAGAAACTT 3531
LOC_Os11g38520.1 AGCATCAGTGGAGCCAAGCATCTTTTTCCATTGTTGACCAAAGGTGGTAATAAGAAACTT 3537
                 *************************************** ********************

A58              GCCAAGGTAACTCTAAGCAACACCCCACTGAACCAAGATGATCTGAAGTTCTTTGCCCAG 3518
W107             GCCAAGGTAACTCTAAGCAACACCCCACTGAACCAAGATGATCTGAAGTTCTTTGCCCAG 3600
Nipponbare       GCCAAGGTAACTCTAAGCAACACCCCACTGAACCAAGATGATCTGAAGTTCTTTGCCCAG 3591
LOC_Os11g38520.1 GCCAAGGTAACTCTAAGCAACACCCCACTGAACCAAGATGATCTGAAGTTCTTTGCCCAG 3597
                 ************************************************************

A58              CTGCCCATGTTACAGTGTGTTAGGCTCCGACACATTTCATGCACCGAGAGTGTGCTCAAC 3578
W107             CTGCCCATGTTACAGTGTGTTAGGCTCCGACACATTTCATGCACCGAGAGTGTGCTCAAC 3660
Nipponbare       CTGCCCATGTTACAGTGTGTTAGGCTCCGACACATTTCATGCACCGAGAGTGTGCTCAAC 3651
LOC_Os11g38520.1 CTGCCCATGTTACAGTGTGTTAGGCTCCGACACATTTCATGCACCGAGAGTGTGCTCAAC 3657
                 ************************************************************

A58              TTCAAGAAAGATGATTTCAAATGCCTCAAGTACCTTCTTATTGAGGGCTCAAACTTGACT 3638
W107             TTCAAGAAAGATGATTTCAAATGCCTCAAGTACCTTCTTATTGAGGGCTCAAACTTGACT 3720
Nipponbare       TTCAAGAAAGATGATTTCAAATGCCTCAAGTACCTTCTTATTGAGGGCTCAAACTTGACT 3711
LOC_Os11g38520.1 TTCAAGAAAGATGATTTCAAATGCCTCAAGTACCTTCTTATTGAGGGCTCAAACTTGACT 3717
                 ************************************************************
```

```
A58                 AATATTACTTTTGAGGATGAGGCAGCCTGTGAGCTCGAGAAGATGGTTTTATCTTCCACT 3698
W107                AATATTACTTTTGAGGATGAGGCAGCCTGTGAGCTCGAGAAGATGGTTTTATCTTCCACT 3780
Nipponbare          AATATTACTTTTGAGGATGAGGCAGCCTGTGAGCTCGAGAAGATGGTTTTATCTTCCACT 3771
LOC_Os11g38520.1    AATATTACTTTTGAGGATGAGGCAGCCTGTGAGCTCGAGAAGATGGTTTTATCTTCCACT 3777
                    ************************************************************

A58                 TGCATAGAGTCTATTTCTGGAGTTCATGGGCTTCCAAAATTCGAAGAGCTTGAGTTGAAC 3758
W107                TGCATAGAGTCTATTTCTGGAGTTCATGGGCTTCCAAAATTCGAAGAGCTTGAGTTGAAC 3840
Nipponbare          TGCATAGAGTCTATTTCTGGAGTTCATGGGCTTCCAAAATTCGAAGAGCTTGAGTTGAAC 3831
LOC_Os11g38520.1    TGCATAGAGTCTATTTCTGGAGTTCATGGGCTTCCAAAATTCGAAGAGCTTGAGTTGAAC 3837
                    ************************************************************

A58                 AGCAGCAGCTGCGGAAGGTTGCTATCATCATGTTTTTACAATGTTGAACGAATAGCCAAG 3818
W107                AGCAGCAGCTGCGGAAGGTTGCTATCATCATGTTTTTACAATGTTGAACGAATAGCCAAG 3900
Nipponbare          AGCAGCAGCTGCGGAAGGTTGCTATCATCATGTTTTTACAATGTTGAACGAATAGCCAAG 3891
LOC_Os11g38520.1    AGCAGCAGCTGCGGAAGGTTGCTATCATCATGTTTTTACAATGTTGAACGAATAGCCAAG 3897
                    ************************************************************

A58                 CTGACTCTTCGTGGTACATTGCTGAAGCAAGGTGATCTACGAATCATCGCCAGGGAACTA 3878
W107                CTGACTCTTCGTGGTACATTGCTGAAGCAAGGTGATCTACGAATCATCGCCAGGGAACTA 3960
Nipponbare          CTGACTCTTCGTGGTACATTGCTGAAGCAAGGTGATCTACGAATCATCGCCAGGGAACTA 3951
LOC_Os11g38520.1    CTGACTCTTCGTGGTACATTGCTGAAGCAAGGTGATCTACGAATCATCGCCAGGGAACTA 3957
                    ************************************************************

A58                 AATATATGCTGCCTAGTGCTCTTGGAAAACTCTTTTGACATAAGCCAGAACCAGATTACC 3938
W107                AATATATGCTGCCTAGTGCTCTTGGAAAACTCTTTTGACATAAGCCAGAACCAGATTACC 4020
Nipponbare          AATATATGCTGCCTAGTGCTCTTGGAAAACTCTTTTGACATAAGCCAGAACCAGATTACC 4011
LOC_Os11g38520.1    AATATATGCTGCCTAGTGCTCTTGGAAAACTCTTTTGACATAAGCCAGAACCAGATTACC 4017
                    ************************************************************

A58                 TTCGAAAAAGAAGAGTTCATATGGCTCAAACTTCTTAGTGTTGATTGCTCTACCATCACC 3998
W107                TTCGAAAAAGAAGAGTTCATATGGCTCAAACTTCTTAGTGTTGATTGCTCTACCATCACC 4080
Nipponbare          TTCGAAAAAGAAGAGTTCATATGGCTCAAACTTCTTAGTGTTGATTGCTCTACCATCACC 4071
LOC_Os11g38520.1    TTCGAAAAAGAAGAGTTCATATGGCTCAAACTTCTTAGTGTTGATTGCTCTACCATCACC 4077
                    ************************************************************

A58                 AAGATTAACTTCATCACTGGATCAGCTCCTAGGCTCAAGAAGATCGTCTGGTCATCTTTC 4058
W107                AAGATTAACTTCATCACTGGATCAGCTCCTAGGCTCAAGAAGATCGTCTGGTCATCTTTC 4140
Nipponbare          AAGATTAACTTCATCACTGGATCAGCTCCTAGGCTCAAGAAGATCGTCTGGTCATCTTTC 4131
LOC_Os11g38520.1    AAGATTAACTTCATCACTGGATCAGCTCCTAGGCTCAAGAAGATCGTCTGGTCATCTTTC 4137
                    ************************************************************

A58                 ACCTCTCTCTCTGGCATCAACAACCTTCCTAGGTTGAAGGAGCTCGAGTTCAATGGATAC 4118
W107                ACCTCTCTCTCTGGCATCAACAACCTTCCTAGGTTGAAGGAGCTCGAGTTCAATGGATAC 4200
Nipponbare          ACCTCTCTCTCTGGCATCAACAACCTTCCTAGGTTGAAGGAGCTCGAGTTCAATGGATAC 4191
LOC_Os11g38520.1    ACCTCTCTCTCTGGCATCAATAACCTTCCTAGGTTGAAGGAGCTCGAGTTCAATGGATAC 4197
                    ******************** ***************************************

A58                 TCAGTCCCAAATGATGTGGAAGAAGCCATTAAAAACAATAAAAGCATAAATCTTAAACAT 4178
W107                TCAGTCCCAAATGATGTGGAAGAAGCCATTAAAAACAATAAAAGCATAAATCTTAAACAT 4260
Nipponbare          TCAGTCCCAAATGATGTGGAAGAAGCCATTAAAAACAATAAAAGCATAAATCTTAAACAT 4251
LOC_Os11g38520.1    TCAGTCCCAAATGATGTGGAAGAAGCCATTAAAAACAATAAAAGCATAAATCTTAAACAT 4257
                    ************************************************************
```

```
A58                   AATAAACCATAA 4190
W107                  AATAAACCATAA 4272
Nipponbare            AATAAACCATAA 4263
LOC_Os11g38520.1      AATAAACCATAA 4269
                      ************
```
