# Supplementary material for: Genetics of chilling response at early growth stage in rice: a recessive gene for tolerance and importance of acclimation
Source: AoB Plants. 2023 Nov 8;15(6):plad075. doi: 10.1093/aobpla/plad075 (PMC10676198; doi:10.1093/aobpla/plad075)
Supplement: plad075_suppl_Supplementary_Figures_S4 [file plad075_suppl_supplementary_figures_s4.pdf]

Fig. S4. Comparison of amino acid sequences of candidate gene for the *ctp-1* locus among A58, W107 and ‘Nipponbare’ (A) and between W107 and ‘Nipponbare’ (B). Amino acid sequence of ‘Hoshinoyume’ (HY) is the same as ‘Nipponbare.’

(A)

```
A58             MTELAAGAVSSLLVVIRNEAVLLGGVRDDVQFIKEEMESMNSFLGHLARSAPQGGEHDEQ   60
W107            MTELAAGAVSSLLVVIRNEAVLLGGVRDDVQFIKEEMESMNSFLGHLARSAPQGGEHDEQ   60
Nipponbare      MTELAAGAVSSLLVVIRNEAVLLGGVRDDVQFIKEEMESMNSFLGHLARSAPQGGEHDEQ   60
                ************************************************************

A58             VRTWMNQVRLLAQDCNNCIDLYLYSGNPEIHRAKGRLRRHLWWVYWSLRKMVAQHRAAIQ   120
W107            VRTWMNQVRLLAQDCNNCIDLYLYSGNPEIHRTKGRLRRHLWWVYWSLRKMVAQHRAAIQ   120
Nipponbare      VRTWMNQVRLLAQDCNNCIDLYLYSGNPEIHRAKGRLRRHLWWVYWSLRKMVAQHRAAIQ   120
                ********************************:***************************

A58             LRQLKDRARDVGERRLRYGVEIPATTKAAPDATGGYAAGDDEEEDEDDREGQFAVATPT   180
W107            LRQLKDRARDVGERRLRYGVEIPATTKAAPDATGGYVAGDDEEEDEDDREGQFAVATPT   180
Nipponbare      LRQLKDRARDVGERRLRYGVEIPATTKAAPDATGGYAAGDDEEEDEDDREGQFAVATPT   180
                ************************************.***********************

A58             LAHHSARWPVFEPPSLDDYVKAKLLEWIEGVPGNAIVTLSIAIVAPDADNKEVLAIAHET   240
W107            LAHHSARWPVFEPPSLDDYVKAKLLEWIGGVPGNAIVTLSIAIVAPDADNKEVLAIAHET   240
Nipponbare      LAHHSARWPVFEPPSLDDYVKAKLLEWIEGVPGNAIVTLSIAIVAPDADNKEVLAIAHET   240
                **************************** *******************************

A58             LVAPDYYYRRSIMVNVPAVHLDFLPLRPKEVLYYILRELEREEAAGSQKQPTDQGEWEEE   300
W107            LVAPNYYYRRSIMVNVPAVHLDFLPLRPKEVLYYILRELEREEAAGSQKQPTDQGEWEEE   300
Nipponbare      LVAPDYYYRRSIMVNVPAVHLDFLPLRPKEVLYYILRELEREEAAGSQKQPTDQGEWEEE   300
                ****:*******************************************************

A58             DPDPWQDYYKKCGIYRSKKGVLDKIKRNIKKMNIYEKLDKIKSDIREGQHKSGKLLLLKL   360
W107            DPDPWQDYYKKCGIYRSKKSVLGKIKRNIKKMNIYEKLDKIKSDIREGQHKSDKLLLLQL   360
Nipponbare      DPDPWQDYYKKCGIYRSKKGVLDKIKRNIKKMNIYEKLDKIKSDIREGQHKSGKLLLLKL   360
                *******************.**.*****************************.*****:*

A58             QEKGADQVDLHVLLQLLVLQSQQDQAKNKAVDTHKLPEWNDNLIEKLAMRLKDHMEADEK   420
W107            QKKGADQVDLHVLLQLLVLQSQQDQAKNKAVDTHKLPEWNDNLIEKLAMRLKDHMEADEK   420
Nipponbare      QEKGADQVDLHVLLQLLVLQSQQDQAKNKAVDTHKLPEWNDNLIEKLAMRLKDHMEADEK   420
                *:**********************************************************

A58             TKKLNEQTGVEEETAVRQGGGGERKEDEKDERGDGEEEGKEERRDMEKG-EERKEQQQEE   479
W107            TKKLNEQTGVEEETAVRQGGGGEREEDEKDERGDGEEEGKEERRDMEKGGEERKEQQQEE   480
Nipponbare      TKKLNEQTGVEEETAVRQGGGGERKEDEKDERGDGEEEGKEERRDMEKG-EERKEQQQEE   479
                ************************:************************ **********

A58             QEKEGRKEEQNEVRKETEGRKEQVAGEEEEKEDHDADNDEDSNDDDDDDEE--EEEDDDD   537
W107            QEKEGRKEEQNEVRKETEGRKEQVAGEEEEKEDHDADNDEDSNDDDDDEEEEEEEEDDND   540
Nipponbare      QEKEGRKEEQNEVRKETEGRKEQVAGEEEEKEDHDADNDEDSNDDDDDEEE--EEEDDDD   537
                ************************************************:**  *****:*
```

```
A58             EEEPIHLHEDQYEQILREVFTKNASSKAQEQDKLVAEQATKTAATTLDEERIKQMIYRFH   597
W107            EEEPIHLHEDQYEQILREVFTKNASSKAQEQDKLVAEQATKTAATTLDEERIKQMINEAK   600
Nipponbare      EEEPIHLHEDQYEQILREVFTKNASSKAQEQDKLVAEQATKTAATTLDEERIKQMINEAK   597
                ******************************************************** . :

A58             LIRIKQQVN---------------------------------------------------   606
W107            QDVLRELRGRETDKNQATGEPDVPPDKNQATGQHAVVLDQNEEAYFEEVEQKIEEIKQEL   660
Nipponbare      QDVLRELRGRETDKNQATGEPDVPPDKNQATGQHAVVLDQNEEAYFEEVEQKIEEIKQEL   657
                   :::  .

A58             ---MQLFWIKTKRL---------ILKK*--------------------------------   621
W107            KEQLKIKWIVDKIKHHLQDQCPLIILKFDQMMDGSRWEEIRKALSLLELSADALIFTTGS   720
Nipponbare      KEQLKIKWIVDKIKHHLQDQCPLIILKFDQMMDGSRWEEIRKALSLLELSADALIFTTGS   717
                   ::: **  *           *: *

A58             ------------------------------------------------------------   621
W107            TEQAKGYCYPPREPIDHCSLVGLYYYTVLKLTSKHKNEDNDNAQIFRGILEECEGHEFCM   780
Nipponbare      TEQAKGYCYPPREPIDHCSLVGLYYYTVLKLTSKHKNEDNDNAQIFRGILEECEGHEFCM   777

A58             ------------------------------------------------------------   621
W107            KIFTHAVYANPKRSNEELRKLHSTLQSPKKSFDTIAKKMFMYSYNDLPKEYKSCLLYLAI   840
Nipponbare      KIFTHAVYANPKRSNEELRKLHSTLQSPKKSFDTIAKKMFMYSYNDLPKEYKSCLLYLAI   837

A58             ------------------------------------------------------------   621
W107            FPKGQKIRRSTLIARWVAEGLTFKEDWPSSVYQANRCFDALIRRWLVYPDDISATGKIKS   900
Nipponbare      FPKGQKIRRSTLIARWVAEGLTFKEDWPSSVYQANRCFDALIRRWLVYPDDISATGKIKS   897

A58             ------------------------------------------------------------   621
W107            CVVGDPVHGFITAIARKQHIVETRLSHHLARHFSIFNDLRLRSSDRIGTFFQGLSRSSRV   960
Nipponbare      CVVGDPVHGFITAIARKQHIVETRLSHHLARHFSIFNDLRLRSSDRIGTFFQGLSRSSRV   957

A58             ------------------------------------------------------------   621
W107            SLLKVLDLEGCQCFASKNQRYLKDICNKMLLLKYLSLKGTDITQLPKEINCLRELEVLDI   1020
Nipponbare      SLLKVLDLEGCQCFASKNQRYLKDICNKMLLLKYLSLKGTDITQLPSEINCLRELEVLDI   1017

A58             ------------------------------------------------------------   621
W107            RETKVPANATVHVLLLKLKRLLAGASQNDPTPRNFVTNVRIPSRIDKMINIEVLSNVKAQ   1080
Nipponbare      RETKVPANATVNVLLLKLKRLLAGASQIDPTPRNFVTNVRIPSRIDKMINIEVLSNVKAQ   1077

A58             ------------------------------------------------------------   621
W107            HHDNLEDIGKLCQLRKLVVVIDGKKSHLGSLLKAISDLHASLRSLSITIPTTTLEVTPSS   1140
Nipponbare      HHDNLEDIGKLCQLRKLGVVIDGKKSHLGSLLKAISDLHASLRSLSITIPTTTLEVTPSS   1137

A58             ------------------------------------------------------------   621
W107            PELQDIASRLKHHPEFLESLSISGAKHLFPLLTEGGNKKLAKVTLSNTPLNQDDLKFFAQ   1200
Nipponbare      PELQDIASRLKHHPEFLESLSISGAKHLFPLLTEGGNKKLAKVTLSNTPLNQDDLKFFAQ   1197
```

```
A58           ------------------------------------------------------------ 621
W107          LPMLQCVRLRHISCTESVLNFKKDDFKCLKYLLIEGSNLTNITFEDEAACELEKMVLSST 1260
Nipponbare    LPMLQCVRLRHISCTESVLNFKKDDFKCLKYLLIEGSNLTNITFEDEAACELEKMVLSST 1257

A58           ------------------------------------------------------------ 621
W107          CIESISGVHGLPKFEELELNSSSCGRLLSSCFYNVERIAKLTLRGTLLKQGDLRIIAREL 1320
Nipponbare    CIESISGVHGLPKFEELELNSSSCGRLLSSCFYNVERIAKLTLRGTLLKQGDLRIIAREL 1317

A58           ------------------------------------------------------------ 621
W107          NICCLVLLENSFDISQNQITFEKEEFIWLKLLSVDCSTITKINFITGSAPRLKKIVWSSF 1380
Nipponbare    NICCLVLLENSFDISQNQITFEKEEFIWLKLLSVDCSTITKINFITGSAPRLKKIVWSSF 1377

A58           -------------------------------------------- 621
W107          TSLSGINNLPRLKELEFNGYSVPNDVEEAIKNNKSINLKHNKP* 1423
Nipponbare    TSLSGINNLPRLKELEFNGYSVPNDVEEAIKNNKSINLKHNKP* 1420
```

(B)

```
W107           MTELAAGAVSSLLVVIRNEAVLLGGVRDDVQFIKEEMESMNSFLGHLARSAPQGGEHDEQ  60
Nipponbare     MTELAAGAVSSLLVVIRNEAVLLGGVRDDVQFIKEEMESMNSFLGHLARSAPQGGEHDEQ  60
               ************************************************************

W107           VRTWMNQVRLLAQDCNNCIDLYLYSGNPEIHRTKGRLRRHLWWVYWSLRKMVAQHRAAIQ  120
Nipponbare     VRTWMNQVRLLAQDCNNCIDLYLYSGNPEIHRAKGRLRRHLWWVYWSLRKMVAQHRAAIQ  120
               ********************************:***************************

W107           LRQLKDRARDVGERRLRYGVEIPATTKAAAPDATGGYVAGDDEEEDEDDREGQFAVATPT  180
Nipponbare     LRQLKDRARDVGERRLRYGVEIPATTKAAAPDATGGYAAGDDEEEDEDDREGQFAVATPT  180
               *************************************.**********************

W107           LAHHSARWPVFEPPSLDDYVKAKLLEWIGGVPGNAIVTLSIAIVAPDADNKEVLAIAHET  240
Nipponbare     LAHHSARWPVFEPPSLDDYVKAKLLEWIEGVPGNAIVTLSIAIVAPDADNKEVLAIAHET  240
               **************************** *******************************

W107           LVAPNYYYRRSIMVNVPAVHLDFLPLRPKEVLYYILRELEREEAAGSQKQPTDQGEWEEE  300
Nipponbare     LVAPDYYYRRSIMVNVPAVHLDFLPLRPKEVLYYILRELEREEAAGSQKQPTDQGEWEEE  300
               ****:*******************************************************

W107           DPDPWQDYYKKCGIYRSKKSVLGKIKRNIKKMNIYEKLDKIKSDIREGQHKSDKLLLLQL  360
Nipponbare     DPDPWQDYYKKCGIYRSKKGVLDKIKRNIKKMNIYEKLDKIKSDIREGQHKSGKLLLLKL  360
               *******************.**.*****************************.*****:*

W107           QKKGADQVDLHVLLQLLVLQSQQDQAKNKAVDTHKLPEWNDNLIEKLAMRLKDHMEADEK  420
Nipponbare     QEKGADQVDLHVLLQLLVLQSQQDQAKNKAVDTHKLPEWNDNLIEKLAMRLKDHMEADEK  420
               *:**********************************************************

W107           TKKLNEQTGVEEETAVRQGGGGEREEDEKDERGDGEEEGKEERRDMEKGGEERKEQQQEE  480
Nipponbare     TKKLNEQTGVEEETAVRQGGGGERKEDEKDERGDGEEEGKEERRDMEKG-EERKEQQQEE  479
               ************************:************************ **********

W107           QEKEGRKEEQNEVRKETEGRKEQVAGEEEEKEDHDADNDEDSNDDDDDEEEEEEEEEDDND  540
Nipponbare     QEKEGRKEEQNEVRKETEGRKEQVAGEEEEKEDHDADNDEDSNDDDDDEEE--EEEDDDD  537
               ***************************************************  *****:*

W107           EEEPIHLHEDQYEQILREVFTKNASSKAQEQDKLVAEQATKTAATTLDEERIKQMINEAK  600
Nipponbare     EEEPIHLHEDQYEQILREVFTKNASSKAQEQDKLVAEQATKTAATTLDEERIKQMINEAK  597
               ************************************************************

W107           QDVLRELRGRETDKNQATGEPDVPPDKNQATGQHAVVLDQNEEAYFEEVEQKIEEIKQEL  660
Nipponbare     QDVLRELRGRETDKNQATGEPDVPPDKNQATGQHAVVLDQNEEAYFEEVEQKIEEIKQEL  657
               ************************************************************


W107           KEQLKIKWIVDKIKHHLQDQCPLIILKFDQMMDGSRWEEIRKALSLLELSADALIFTTGS  720
Nipponbare     KEQLKIKWIVDKIKHHLQDQCPLIILKFDQMMDGSRWEEIRKALSLLELSADALIFTTGS  717
               ************************************************************

W107           TEQAKGYCYPPREPIDHCSLVGLYYYTVLKLTSKHKNEDNDNAQIFRGILEECEGHEFCM  780
Nipponbare     TEQAKGYCYPPREPIDHCSLVGLYYYTVLKLTSKHKNEDNDNAQIFRGILEECEGHEFCM  777
               ************************************************************
```

```
W107            KIFTHAVYANPKRSNEELRKLHSTLQSPKKSFDTIAKKMFMYSYNDLPKEYKSCLLYLAI   840
Nipponbare      KIFTHAVYANPKRSNEELRKLHSTLQSPKKSFDTIAKKMFMYSYNDLPKEYKSCLLYLAI   837
                ************************************************************

W107            FPKGQKIRRSTLIARWVAEGLTFKEDWPSSVYQANRCFDALIRRWLVYPDDISATGKIKS   900
Nipponbare      FPKGQKIRRSTLIARWVAEGLTFKEDWPSSVYQANRCFDALIRRWLVYPDDISATGKIKS   897
                ************************************************************

W107            CVVGDPVHGFITAIARKQHIVETRLSHHLARHFSIFNDLRLRSSDRIGTFFQGLSRSSRV   960
Nipponbare      CVVGDPVHGFITAIARKQHIVETRLSHHLARHFSIFNDLRLRSSDRIGTFFQGLSRSSRV   957
                ************************************************************

W107            SLLKVLDLEGCQCFASKNQRYLKDICNKMLLLKYLSLKGTDITQLPKEINCLRELEVLDI   1020
Nipponbare      SLLKVLDLEGCQCFASKNQRYLKDICNKMLLLKYLSLKGTDITQLPSEINCLRELEVLDI   1017
                **********************************************.*************

W107            RETKVPANATVHVLLLKLKRLLAGASQNDPTPRNFVTNVRIPSRIDKMINIEVLSNVKAQ   1080
Nipponbare      RETKVPANATVNVLLLKLKRLLAGASQIDPTPRNFVTNVRIPSRIDKMINIEVLSNVKAQ   1077
                ***********:*************** ********************************

W107            HHDNLEDIGKLCQLRKLVVVIDGKKSHLGSLLKAISDLHASLRSLSITIPTTTLEVTPSS   1140
Nipponbare      HHDNLEDIGKLCQLRKLGVVIDGKKSHLGSLLKAISDLHASLRSLSITIPTTTLEVTPSS   1137
                ***************** ******************************************

W107            PELQDIASRLKHHPEFLESLSISGAKHLFPLLTEGGNKKLAKVTLSNTPLNQDDLKFFAQ   1200
Nipponbare      PELQDIASRLKHHPEFLESLSISGAKHLFPLLTEGGNKKLAKVTLSNTPLNQDDLKFFAQ   1197
                ************************************************************

W107            LPMLQCVRLRHISCTESVLNFKKDDFKCLKYLLIEGSNLTNITFEDEAACELEKMVLSST   1260
Nipponbare      LPMLQCVRLRHISCTESVLNFKKDDFKCLKYLLIEGSNLTNITFEDEAACELEKMVLSST   1257
                ************************************************************

W107            CIESISGVHGLPKFEELELNSSSCGRLLSSCFYNVERIAKLTLRGTLLKQGDLRIIAREL   1320
Nipponbare      CIESISGVHGLPKFEELELNSSSCGRLLSSCFYNVERIAKLTLRGTLLKQGDLRIIAREL   1317
                ************************************************************

W107            NICCLVLLENSFDISQNQITFEKEEFIWLKLLSVDCSTITKINFITGSAPRLKKIVWSSF   1380
Nipponbare      NICCLVLLENSFDISQNQITFEKEEFIWLKLLSVDCSTITKINFITGSAPRLKKIVWSSF   1377
                ************************************************************

W107            TSLSGINNLPRLKELEFNGYSVPNDVEEAIKNNKSINLKHNKP* 1423
Nipponbare      TSLSGINNLPRLKELEFNGYSVPNDVEEAIKNNKSINLKHNKP* 1420
                *******************************************
```
