# Supplementary material for: Genetics of chilling response at early growth stage in rice: a recessive gene for tolerance and importance of acclimation
Source: AoB Plants. 2023 Nov 8;15(6):plad075. doi: 10.1093/aobpla/plad075 (PMC10676198; doi:10.1093/aobpla/plad075)
Supplement: plad075_suppl_Supplementary_Figures_S5 [file plad075_suppl_supplementary_figures_s5.pdf]

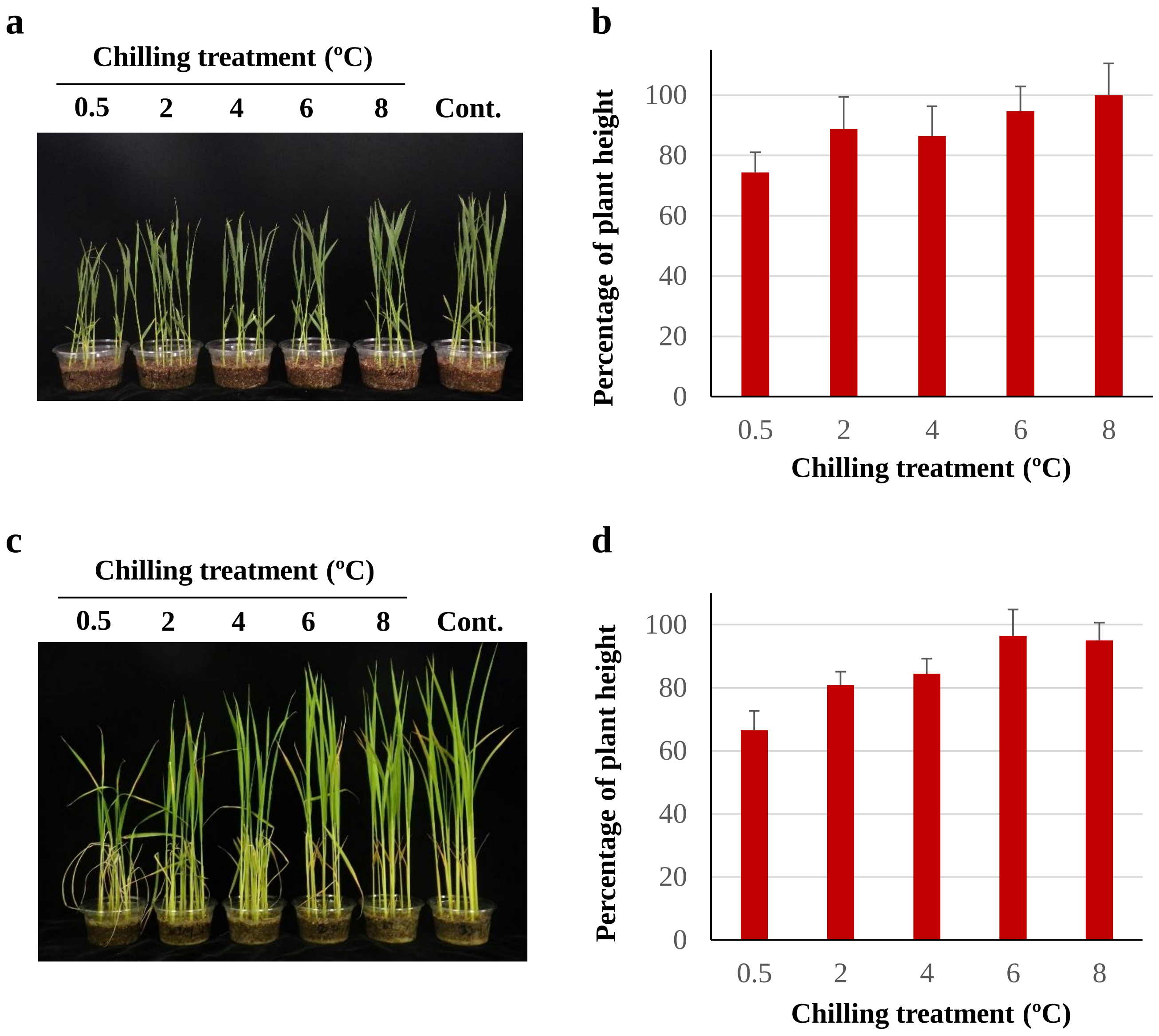

Fig. S5. Chilling tolerance of J501 under different chilling temperature condition at the plumule (**a** and **b**) and seedling stage (**c** and **d**). Plant height 10 d after chilling treatment at plumule stage (**a**) and two weeks after chilling treatment at seedling stage (**c**). The degree of chilling tolerance at plumule (**b**) and seedling (**d**) stages was evaluated as the percentage of plant height as described in Materials and Methods.
